# Gaming Disorder: The role of a gamers flow profile

**DOI:** 10.1016/j.abrep.2024.100555

**Published:** 2024-06-02

**Authors:** Trent Footitt, Natasha Christofi, Dylan R Poulus, Michelle Colder Carras, Vasileios Stavropoulos

**Affiliations:** a Department of Psychology, Applied Health, School of Health and Biomedical Sciences, RMIT University, Melbourne, Australia; b Physical Activity, Sport, and Exercise Research Theme, Faculty of Health, Southern Cross University, Australia; c Johns Hopkins Bloomberg School of Public Health, Baltimore, United States

**Keywords:** Internet Gaming Disorder, Latent Profile Analysis, Online Flow

## Abstract

Digital games are widely popular and integral to contemporary entertainment. Nevertheless, a proportion of users present with disordered/excessive gaming behaviours, provisionally classified as Internet Gaming Disorder (IGD). Previous literature suggests examining the contribution of an individual’s profile of immersive engagement with their gaming activity, known as online flow, for disordered gaming behaviours. Therefore, the main goals of this study were (1) to categorise gamers into distinct profiles based on their online flow experiences and (2) to investigate the differences in disordered gaming among these different flow profiles. A sample of 565 gamers (12–68 years, Mage = 29.3 years) was assessed twice over six months with the Online Flow Questionnaire (OFQ), the Internet Gaming Disorder Scale-Short-Form (IGDS9-SF), and the Gaming Disorder Test (GDT). Latent profile analysis (LPA) identified five distinct profiles encompassing ’High-Flow with High Loss of Control’ (HF-HLOC; 14.0 %), ’Low Flow with Low Enjoyment’ (LF-LE; 11.9 %), ’Average Flow with Low Enjoyment’ (AF-LE; 17.5 %), ’Low Flow with High Enjoyment’ (LF-HE; 20.2 %), and ’High Loss of Sense of Time with Low Loss of Control’ groups (HLOT-LLOC; 36.5 %). As hypothesised, individuals across varying profiles evidenced differences in their concurrent and longitudinal disordered gaming behaviours. Overall, findings suggest that ’loss of sense of time’ may be the most pivotal factor in differentiating flow states and profiles during gaming, advocating its consideration in disordered gaming assessment and treatment.

## Highlights

• Five gamer flow profiles identified, influencing gaming disorder.
• ’Loss of time’ crucial in differentiating gamer flow states.
• High-flow gamers show increased disordered gaming risks.
• Flow profiles linked to distinct gaming disorder behaviours.

## 1 Gaming Disorder: The role of a gamers flow profile

Digital games are an integral form of contemporary entertainment for younger people (Paulus et al., 2018). Not surprisingly, for most players, gaming is regarded as an engaging and stimulating leisure-time activity (Loton et al., 2016). Within the context of the proliferation and advancements of digital technology (e.g., broadly accessible mobile gamified apps) during the last decade, internet gaming has seen a further surge in its popularity, currently exceeding three billion users across the globe (Newzoo, 2022). Alongside widespread growth, there has been great variability in the intensity of involvement with online games (e.g., low, moderate, excessive; (Colder Carras et al., 2021). These have prompted researchers to investigate the potential positive and negative impacts of both moderate and excessive internet gaming patterns on an individual's well-being (Kim et al., 2022, Stavropoulos et al., 2018a, Stavropoulos et al., 2019a). Research into gamers' well-being has emphasised the various social, emotional, and health advantages of moderate/healthy gaming engagement (e.g. gaining satisfaction, making new friends, fine-motor skill development, and psychological skill development; (Granic et al., 2014, Raith et al., 2021, Trotter et al., 2021), while other studies have focused on the emerging risks of excessive/unhealthy/addictive/disordered gaming, and its clinically significant consequences (e.g. compromised educational and employment performance, as well as personal and romantic relationships; (Anderson et al., 2017, Stavropoulos et al., 2018b, Stavropoulos et al., 2021). The current study explores excessive gaming behaviours in relation to the profile of an individual's absorbance by what they are doing online (i.e. gaming activity), often described as online flow (Stavropoulos et al., 2021).

### 1.1 Disordered gaming

A significant amount of research has recently emerged exploring the potential classification of problematic gaming as a formal disorder (Blake and Sauermilch, 2021, Stavropoulos et al., 2020, American Psychiatric Association, 2013). As a result, Internet Gaming Disorder (IGD) was included in the fifth edition of the *Diagnostic and Statistical Manual of Mental Disorders* (DSM-5) in 2013, with its status as an independent disorder remaining contingent on additional clinical and research evidence (APA, 2013). Similarly, in 2019, the World Health Organisation (WHO) formalised Gaming Disorder (GD) as an official classification in the 11th edition of the International Classification of Diseases (ICD-11; WHO, 2019).

Specifically, the DSM-5 proposes nine criteria for diagnosing IGD, of which a minimum of five symptoms must be present throughout 12 months (APA, 2013). These criteria include 1: a high preoccupation with video games (preoccupation); 2: withdrawal symptoms (e.g. irritability and frustration when one is not gaming); 3: an increased tolerance to video gaming (i.e. requiring a progressively extended duration of gameplay to attain satisfaction; tolerance); 4: inability to stop or limit gaming despite wishing/aiming the opposite (loss of control), 5: a loss of interest in activities and/or engagement with people that were once enjoyable (loss of interest); 6: persistent gaming despite adverse consequences (continuation); 7: deception about a player's engagement in gaming activities to others (e.g. intentionally lying about gameplay time and/or related expenses; deception); 8: utilising games as a means of escape from negative moods (e.g. a player initially engages with the game to feel better and to gradually to feel less worse; escape/ mood modification); and 9: impairment of relationships, work, or educational opportunities as a result of excessive gaming (functional impairment; (Kovacs et al., 2022, Morcos et al., 2021).

The WHO (2019) described disordered gaming via only three core diagnostic behaviours (Pontes et al., 2020). These criteria include (1) loss of control over gaming (e.g., higher frequency, intensity, and duration than what an individual would consciously aim); (2) prioritisation of gaming over other life interests and daily activities (e.g., an individual prefers gaming than working, studying and/or socialising); (3) persistent and/or escalated engagement with gaming despite the occurrence of negative consequences (e.g., loss of employment/income; exams/assignment failures; isolation from others (Kircaburun et al., 2020). Given that the two definitions (i.e., American Psychiatric Association, 2013, World Health Organization, 2019) have been found to operate with some differences (e.g., the proportions of those identified as being at risk for disordered gaming present dissimilar (Pontes et al., 2019), in the current project both sets of criteria were chosen to be concurrently employed to: a) enhance comparability with available international empirical evidence; and b) adhere to scholars recommendations for consistency in the field (Stavropoulos et al., 2019b).

### 1.2 Aetiology of disordered gaming

Despite the different diagnostic definitions, several theoretical frameworks aiming to explain disordered gaming appear to agree that disordered gaming usage involves individual, surrounding, and game-related factors (Anderson et al., 2017, Brand et al., 2016: Stavropoulos et al., 2021). While holistic models have been developed to understand disordered gaming, and progress has been made in identifying the various individual risk factors (e.g., higher levels of anxiety, depression, and lower physical activity; [Adams et al., 2019, Burleigh et al., 2018, Liew et al., 2018]), some scholars call for greater attention to be given to game-activity related factors (i.e., an individual's engagement by what they are doing online; (Hu et al., 2019, Stavropoulos et al., 2019a). Specifically, game-activity factors like the absorbing/addictive components of digital games, may captivate vulnerable players (Hu et al., 2019, Stavropoulos et al., 2019a). Indeed, two major hypotheses have been proposed to elucidate the interaction between individual vulnerabilities and attractive/immersive game elements that contribute to disordered gaming (Brand et al., 2016, Griffiths et al., 2017, Kardefelt-Winther et al., 2017). Firstly, the self-medication hypothesis (Khantzian, 1997) has been adapted to explain how problematic online gaming can offer alleviation of negative affect to distressed individuals (i.e., immersive gaming to relieve feelings of anxiety, depression, and stress (Griffiths et al., 2017, Kovacs et al., 2022). In other words, an individual uses excessive/disordered gaming as a way to self-medicate their other pre-existing symptoms (Griffiths et al., 2017, Kovacs et al., 2022). Secondly, the Compensatory Internet Use (CIU) model proposes that excessive gaming may act as an adaptive mechanism for individuals to cope and/or reduce psychopathological symptoms or adverse life events via their gratifying in-game experiences (Kardefelt-Winther et al., 2017). In the CIU model, a user's absorbance in their gaming world/surrounding (e.g., experiencing the latter as real, to the extent that they feel as if they are actually there) has been defined as “presence”, while their absorbance by their levelled gaming activity (e.g., progressively higher game challenges) has been broadly defined as “game flow” (Stavropoulos et al., 2021). Gaming flow (i.e., immersive activity) has been demonstrated to have a greater influence on one's excessive digital game usage and is often exacerbated by a player's stronger sense of presence (i.e., immersive online context; Kiatsakared & Chen, 2022). Due to the potential influence of flow on disordered gaming, recent research has called for investigating potentially distinct flow profiles as contributors to the development of disordered gaming (Stavropoulos et al., 2022). In particular, over-engagement with gaming, due to the levelled structure of modern games, has been supported to increase flow via progressively higher levels of challenges, inviting, in turn, disordered usage (Stavropoulos et al., 2021, Stavropoulos et al., 2022).

Investigating the relationship between flow and disordered gaming aligns with the alternative 'typological' model advocated by many researchers to gain deeper insight into the different manifestations of disordered gaming (Billieux et al., 2015, Colder Carras and Kardefelt-Winther, 2018, Lee et al., 2017; Tullet-Prado et al., 2021). The alternative typological model is mainly centred around identifying the differences across various gamer profiles to provide insight into “who” is actually at risk, and thus moving from a “variable” focused to a more “personalised” research perspective. Instead of addressing gaming flow as a risk factor, the focus shifts to portraying different profiles of users based on the intensity and quality of their flow experiences (Stavropoulos et al., 2020, Stavropoulos et al., 2021). Several studies to date have examined variations among gamers' profiles in terms of their disordered gaming propensity by using latent profile analysis (LPA; [Colder Carras and Kardefelt-Winther, 2018, Lemmens et al., 2015; Tullet-Prado et al., 2021]. For example, Tullet-Prado and colleagues (2021) used the Internet Gaming Disorder Scale-Short-Form (IGDS9-SF) and, via LPA, proposed four distinct disordered gaming classes among a sample of gamers (*n* = 1032). These profiles ranged from 'IGD aversive', 'Normative', 'Moderate IGD risk' to 'High IGD Risk' gamers. Similarly, Lemmens et al. (2015) employed the IGDS9-SF to categorise gamers into three profiles (i.e., normal, risky, disordered) based on variables such as violence, gaming duration, self-esteem, and loneliness. Further, Colder Carras & Kardefelt-Winther (2018) revealed five profiles (e.g., engaged, normative, concerned, at risk, IGD) in a population of 7865 gamers, utilising the Assessment of Internet and Computer game Addiction-Gaming Module. Interestingly, both the “engaged” and “concerned” profiles identified in this research had a significantly higher association with depression and anxiety behaviours reported (Colder Carras & Kardefelt-Winther, 2018). Despite these studies having identified distinct profiles based on disordered gaming characteristics, no study has yet explored the occurrence of flow typologies/profiles and their possible differences regarding the development of disordered gaming (Stavropoulos et al., 2022).

### 1.3 Profiling flow

The degree of involvement in online activity in the context of online flow has been closely linked with an individual's positive affect (e.g., gratification) and disordered usage (Stavropoulos et al., 2018b, Stavropoulos et al., 2021). The concept of flow has been adapted for online gaming research and refers to the optimal level of experience that is reached in gaming (Csikszentmihalyi & Nakamura, 2018). It is described by several different features, including (i) high levels of physiological and psychological engagement in the activity (e.g., one is physically absorbed while gaming); (ii) undivided attention on the activity (e.g. while gaming, one is disconnected by their surroundings and their concurrent obligations); (iii) disregard for the completion of the activity itself (e.g., it is not achieving the game's final goal, rather the gaming journey itself that makes a player satisfied); and (iv) distorted perception of time and time loss (e.g., a player might find themselves playing for several hours without recognising the amount of time they have consumed; Csikszentmihalyi and Nakamura, 2018, Hu et al., 2019). Literature additionally specifies that the in-game flow state is achieved when the individual's level of gaming skill and the game difficulty/challenge posed on them are well-matched (a player's in-game skills are slightly lower than the in-game challenges they are facing; [Csikszentmihalyi and Nakamura, 2018, Hu et al., 2019, Stavropoulos et al., 2018b]). Based on these facets/features, flow is recognised as a powerful intrinsic motivator that helps sustain online behaviour and, for some users, over-engagement and/or disordered gaming (Li et al., 2021).

Nevertheless, one may not necessarily experience the different flow features at the same level/intensity (Stavropoulos et al., 2021, Stavropoulos et al., 2022). Some may experience a higher sense of loss of time, while others may experience higher arousal/absorption and/or disconnection from their surroundings, thus informing different gaming flow profiles that should/could be presenting with varying disordered gaming risk, and thus require tailored interventions (Csikszentmihalyi and Nakamura, 2018, Hu et al., 2019, Li et al., 2021). For example, Hu et al. (2018) explored the association between the preference for social games, online flow, and their contribution to disordered gaming. The results showed that the experience of flow mediated a preference for social games (such as Massively Multiplayer Online Role-Playing Games and Multiplayer Online Battle Arena games) and disordered gaming, implying that it is specifically the competition with others (and not so much other flow features), that leads to one's disconnection from their surrounding experienced in the context of their flow state (Hu et al., 2018). Overall, while the pursuit of online flow (e.g., immersive pleasure) in gaming appears comparable with analogous behaviours seen in other behavioural addictions, the existence of differing intensity and/or features of online flow profiles remains to be assessed (Trivedi & Teichert, 2017). Investigating such distinct flow profiles (i.e., immersive pleasure related to gaming action) appears imperative, as different flow features may possess a different dose–response effect, resulting in varying gaming disorder behaviours. This might also imply that although some gamer profiles may develop a tolerance to certain flow features, leading them to engage in more frequent and extended gaming periods to fulfil their needs, others may not (Stavropoulos et al., 2018b). As a result, different flow profiles of gamers may be differentially susceptible to developing gaming disorder behaviours requiring different tailored prevention and intervention management when addressing the symptom-perpetuating effects of online flow (Hu et al., 2018; Li et al., 2021).

### 1.4 Current study

There is clear evidence of a strong overall association/link between online flow and disordered gaming (Hu et al., 2018; Stavropoulos et al., 2021, Stavropoulos et al., 2022). However, as flow is a multifaceted experience, which assumes a strong sense of game challenge, entails a distorted sense of time and/or disconnection from one's surroundings, and centres on the enjoyment experienced in the activity participation itself, rather than its completion/outcome. Different gamers may experience different flow features at varying levels, thus informing distinct flow profiles with unequal vulnerability to disordered gaming (Hu et al., 2018; Stavropoulos et al., 2021, Stavropoulos et al., 2022). Research has yet to examine the occurrence of specific flow profiles, which may vary regarding disordered gaming risk. Understanding such individual differences considering disordered gaming, particularly in relation to gaming flow, may give significant insight into more effective and personalised prevention and intervention planning (Gorowska et al., 2022).

To the relationship between flow states and disordered gaming, the present study aims to achieve two goals; i) to build upon previous research by examining the typologies/profiles of in-game flow states among a gaming community population. Specifically, this study will investigate whether an online community sample of gamers can be described by different flow typologies/profiles. ii) To explore the role of different flow profiles in the development of disordered gaming and examine whether there is a significant difference in the disordered gaming behaviours manifested between the different flow profiles. The study will use innovative statistical analysis methods, by analysing a substantial longitudinal sample of gamers (N > 500 in wave 1), assessed across two time points, six months apart, and utilising a well-validated and widely used scale to measure the psychometric properties of online Flow (i.e., Online Flow Questionnaire; OFQ; Chen et al., 1999). Additionally, 12 advanced statistical models for profiling will be simultaneously calculated and compared (Rosenberg et al., 2019). To address these specific aims, the following research questions were explored:

**RQ1-** Considering the various behaviours of flow, what is the best way to characterise the sample examined in wave 1 in terms of the number and type of flow profiles?

**RQ2-** What is the size of each profile as described by the different wave 1 flow behaviours as indicators?

In addition, the following hypothesis was explored:

**H1-** Participants classified across different flow profiles are expected to significantly differ regarding their disordered gaming behaviours experienced concurrently (wave 1) and over time (i.e., six months later; wave 2).

## 2 Methods

### 2.1 Participants

An initial number of 627 respondents were recruited. Of those, 7 were excluded due to being preview-only responses, 19 were identified as spam, 1 as a potential bot, 12 did not provide consent, 8 failed validity questions (e.g., claimed to play non-existing games such as “Risk of Phantom”), and 15 had insufficient responses. Therefore, the final sample consisted of 565 adult/adolescent participants (M_age_ = 29.3 years SD = 10.6, Min_age_ = 12 Max_age_ = 68; Males_cisgender_ = 283, 50.1 %) with an up to par maximum sampling error of ± 4.12 % (95 % CI, z = 1.96). These individuals were assessed longitudinally in the community, with a 6-month gap between two time points. Considering their demographics_time_point_1_, 271 (55.3 %) reported being employed full-time, 176 (36 %) held an undergraduate degree, 359 (73.6 %) identified as heterosexual, 410 (72.5 %) identified as having Australian/English ancestry, 142 (25.1 %) resided with their family of origin, and 148 (30.2 %) were single. Considering their gaming patterns _time_point_1_, they reported gaming on average for 5.62 years (Min _gaming-years_= <1 year, Max _gaming-years_ = 30; SD = 4.49). On weekdays, they reported an average of 2.23 h of gaming per day (Min _daily-gaming-time-weekdays_ = <1 h, Max _daily-gaming-time-weekdays_ = 15; SD = 1.82) and during the weekend, they reported 3.39 h of gaming per day (Min _daily-gaming-time-weekend_ = <1 h, Max _daily-gaming-time-weekend_ = 18; SD = 2.40). Considering social media _time_point_1_, they reported usage for an average of 7.06 years (Min _social-media-usage-years_= <1 year, Max _social-media-usage-years_ = 17; SD = 7.06), consuming an average time of 2.55 h on weekdays (Min _daily-social-media-usage-time-weekdays_ = <1 h, Max _daily-social-media-usage-time-weekdays_ = 15; SD = 2.16) and 3.01 h during the weekend (Min _daily-social-media-usage-time-weekend_ = <1 h, Max _daily-social-media-usage-time-weekend_ = 16; SD = 2.48) with 145 (26 %) stating Facebook as their preferred platform. The maximum random sampling error for a sample of 565 at the 95 % confidence interval (z = 1.96) equals +/- 4.12 % satisfying Hill's [46] recommendations. Missing values of analyses variables_time_point_1_ ranged between 4 (0.7% item 1 Internet Gaming Disorder Scale 9 items Short Form) to 51 (2.83 % item 12 item 2 Online Flow Questionnaire) and were missing completely at random in the broader dataset (MCAR_test_ = 38.4, *p* = 0.14 _[9 missing patterns]_; [47]). Attrition between waves was 276 participants (48.8 %) and thus revealed low to moderate effect-sizes regarding gender (x^2^ = 4.26, df = 6, *p* = 0.642, Cramer's V = 0.087), sexual orientation (x^2^ = 7.75, df = 4, *p* = 0.101, Cramer's V = 0.126), ancestry (x^2^ = 8.94, df = 4, *p* = 0.063, Cramer's V = 0.126), romantic relationship engagement (x^2^ = 3.76, df = 4, *p* = 0.440, Cramer's V = 0.088), educational status (x^2^ = 11.2, df = 7, *p* = 0.129, Cramer's V = 0.152), employment status (x^2^ = 7.58, df = 6, *p* = 0.271, Cramer's V = 0.124), gaming years (t_Welch's_ = 3.509, df = 526, *p < 0.001*, Cohen's d = 0.296), average daily gaming time during the week (t_Student's_ = 0.873, df = 555, *p* = 0.383, Cohen's d = -0.0741), average daily gaming time during the weekend (t_Student's_ = 0.159, df = 553, *p* = 0.874, Cohen's d = 0.0135), social media usage years (t_Sudent's_ = 2.501, df = 556, *p =* 0.013, Cohen's d = 0.2118), average daily social media usage time during the week (t_Student's_ = -2.313, df = 543, *p* = 0.021, Cohen's d = -0.1983), average daily social media usage time on the weekend (t_Welch's_ = -2.447, df = 501, *p* = 0.015, Cohen's d = -0.2111) and age (t_Student's_ = 4.967, df = 560, *p < 0.001*, Cohen's d = 0.4192; see Appendix A, Tables 1-7). A description of the sample at time point 1 can be found in Table 1 and Appendix D, Table 11. Data is available online (see Stavropoulos et al., 2023) and has been used to address different research questions in two past published studies (see [Brown et al., 2024, Stavropoulos et al., 2023]).

**Table 1:** Participant's age, game playing years and daily week and weekend time across time point one (T1) and time point two (T2).

	Age (T1)	Daily Game Time in the Week (T1)	Daily Game Time in the Weekend (T1)	Years of Gaming (T1)	Age (T2)	Years of Gaming (T2)	Daily Game Time in the Week (T2)	Daily Game Time in the Weekend (T2)
N	562	557	555	558	289	288	286	285
Mean	29.3	2.23	3.39	7.06	31.9	4.60	2.11	2.88
SD	10.6	1.82	2.40	4.41	10.1	4.96	2.61	2.06
Min	12.0	0.00	0.00	0.00	12.0	0.00	0.00	0.00
Max	68.0	15.0	18.0	17.0	61.0	23.0	23.0	12.0

### 2.2 Measures

Sociodemographic variables, including an individual's age and gender, as well as internet user questions, were included prior to the psychometric scales (see Table 1).

#### 2.2.1 Gaming flow

The Online Flow Questionnaire (OFQ) was used to assess the level of absorption experienced by individuals during online activities (Stavropoulos et al., 2022). The original scale included 5 flow experience “yes” or “no” filter questions, matched with an item addressing the app where this was experienced. The 5th item focused on the control one experienced in the context of balance between their skills and the tasks (Chen et al., 1999). In the revised version used here only the five questions targeting the different aspects of the flow experience (and not the application, where this was encountered, as it addressed gaming for all participants; [Stavropoulos et al., 2022]). Accordingly, item 5 is revised to better reflect the sense of controlling activity absorbance and disconnection a subject may experience while in a state of flow (Stavropoulos et al., 2022). It consists of five, five-point Likert questions, with responses ranging from 0 (*Not at all*) to 4 (*Absolutely*), referring to the experience of the distinct flow states and/or features (e.g., “*Have you ever experienced the feeling of 'being in control' during your Web navigation?”).* The total score was produced by adding relevant item scores ranging between 0 and 20 (across the five items), where higher scores signify greater levels of flow experience. The OFQ demonstrated sufficient internal reliability in both the present study in wave 1 (Cronbach's α = 0.659, Ω McDonald = 0.680) and 2 (Cronbach α = 0.670, Ω McDonald = 0.690).

#### 2.2.2 Internet gaming disorder

Internet Gaming Disorder Scale-Short-Form (IGDS9-SF; [Pontes & Griffiths, 2015) is a short 9-item psychometric continuous (i.e. minimum to maximum) measure of Internet Gaming Disorder behaviours/criteria as enlisted in DSM-5 (APA, 2013). This self-report questionnaire assesses the disordered effects of excessive/problematic online and offline gaming activities over 12 months. The scale consists of items measured on a 5-point Likert scale, ranging from 1 (*Never*) to 5 (*Very Often*). The scale allows for total scores produced by adding the respective 9 items' points, ranging from 9 to 45, where higher scores indicate greater levels of reported IGD behaviours (e.g. “*Do you feel more irritability, anxiety, or even sadness when you try to either reduce or stop your gaming activity?”).* The IGDS9-SF demonstrated satisfactory internal reliability for the current data in wave 1 (Cronbach α = 0.846, Ω McDonald = 0.858) and 2 (Cronbach α = 0.861, Ω McDonald = 0.871).

#### 2.2.3 Gaming disorder

The Gaming Disorder Test (GDT; [22]) is a 4-item psychometric measure of Gaming Disorder (GD) in accordance with the diagnostic criteria developed by the WHO (World Health Organisation, 2019), as seen in the ICD-11. The GDT assesses disordered gaming-related activity carried out from a computer/laptop, various gaming consoles, or any other related devices (e.g., mobile phone, tablet, etc.) during the past year. The four items composing this unidimensional tool are scored on a 5-point Likert scale, with answers ranging from 0 (*Never*) to 4 (*Very Often*). Examples include: *“I have experienced significant problems in life (*e.g., *personal, family, social, education, occupational) due to the severity of my gaming behaviour”.* The total scores are produced by the addition of the relevant item points. They can range from a minimum of 4 to a maximum of 20 points, with higher points signifying a higher degree of disordered gaming behaviours reported. The GDT has demonstrated satisfactory levels of reliability for the current data wave 1 (Cronbach's α = 0.808, Ω McDonalds = 0.812) and 2 (Cronbach α = 0.854, Ω McDonald = 0.862).

### 2.3 Procedure

Approvals for the study were granted by the Human Research Ethics Committee of Victoria University [HRE21-044], the Department of Education and Training of The Victorian State Government [2022_004542], and the Melbourne Archdiocese of Catholic Schools [1179]. The sample participants were collected from various sources in the community, including universities (e.g., RMIT, Victoria, Melbourne, and Deakin Universities ), Australian gamers' groups (e.g., Aus Gaymers Network), venues (e.g., Fortress Melbourne), and online forums (e.g., AusGamers), as well as advertising via *YouTube* videos. Adolescents and adults aged 12 and above could participate voluntarily and anonymously. Participants were required to read a plain language information statement that described their participant rights, the aims of the study, the risks and then provide informed consent. For adolescents participants between 12 and 18 years of age, it was required their responsible parent/guardian to read and complete the plain language statement, followed by the adolescents themselves providing assent. Data collection consisted of three separate data streams linked to each participant through a unique non-identifiable code. These streams included: a) a series of demographic, online activity (including internet, gaming, and social media use), and psychometric assessments/questionnaires. Participants accessed these through an online Qualtrics link, which required completion of the plain language information statement prior to completion. After reviewing the statement, they were requested to provide informed consent by ticking a box, allowing them to commence the survey; b) use an actigraphy device (such as a Fitbit) for a week to track physical activity and sleep patterns, including daily step count and duration of sleep. The information gathered from the Fitbit device was digitally linked to the participants’ other datasets using a unique identifier (i.e., data was automatically retrieved from the Fitbit portal using the participant's specific code). Participants without a Fitbit were given one at meetings organized with the research team. Additionally, participants used a mobile monitoring app named Aware Light (Stavropoulos et al., 2023) for a week. This app tracked details such as screen on/off times, the number and duration of calls, and the length of texts in characters. The data from Aware Light was also synchronized with other datasets using the unique code of each participant. This data collection procedure was to be repeated four times, occurring once every six months between 2021 and 2023. The current study is based on the first two completed collection waves (detailed information can be found in Appendix B).

### 2.4 Statistical analyses

To investigate *RQ1* and *RQ2*, the five online flow subscale items assessed by OFQ were applied as indicators for a sequence of latent profile analyses (LPA) models using the TIDYLPA CRAN package in R (Rosenberg et al., 2019). LPA was guided by its data-driven modelling approach, which enables the identification of naturally homogeneous subgroups (profiles) within a population based on meaningful descriptors or distinctive characteristics (in this case, the different flow items; [Muthén & Muthén, 2016]) The LPA modelling employed a Maximum Likelihood Estimator (MLE) to categorise profile membership likelihoods among gamers based on their gaming flow symptoms. TidyLPA was chosen for its capacity to predict optimal relationships between indicators across differing profiles, such as means (i.e., average levels of reported online flow features), variances (i.e., variability of online flow features, as profile indicators, within the profiles), and covariances (i.e., variability of the reported online flow items' responses, across the profiles identified;). It does so by allowing the assessment of four different model parameterisations (see Appendix D; Masyn, 2013). At this point it should be noted that although there is no minimum recommended sample size for LPA/LCA, simulation investigations tend to support a threshold exceeding 500 participants to proceed, with a higher number of more informative indicators likely compensating for smaller sample sizes (Kongsted and Nielsen, 2017, McLachlan, 1987). Both these recommendations are satisfied in the current analyses.

Determining the optimal number and structure/parameterization of latent profiles involved several steps. Firstly, the best combination of parameters (including non, partially and/or fully constrained profile means, variances, and covariances) was determined by comparing models based on various fit criteria. The criteria included the Akaike Information Criterion (AIC), the Bayesian Information Criterion (BIC), the Approximate Weight of Evidence Criterion (AWE), the Classification Likelihood Criterion (CLC), and the Kullback Information Criterion (KIC), where lower rates/numbers suggest better fit (Masyn, 2013). Secondly, the optimal number of profiles was assessed via the bootstrapped likelihood ratio test (BLRT). BLRT is conducted to determine whether adding an additional latent profile significantly improved fit (with *p* < 0.5 being an indication of better fit; [McLachlan, 1987]). Thus, if the BLRT is insignificant, the model fit does not improve by adding another profile. Lastly, the heterogeneity levels across latent profiles were evaluated by examining the standardised entropy criterion (*h*). An entropy range of 0.40–0.60 is supported to indicate low, 0.60–0.80 medium, and > 0.80 high entropy [Celeux and Soromenho, 1996, Clark and Muthén, 2009].

Finally, to address *H1*, four successive one-way ANOVAs were conducted to evaluate the difference in disordered scores between the online flow profiles revealed as per the IGDS9-SF and the GDT at time points 1 and 2, respectively. Additional post hoc analyses were performed to detail observed differences.

## 3 Results

### 3.1 Identifying and describing flow profiles

To address *RQ1* and *RQ2*, our objective was to determine the most suitable number of latent profiles and the proportion of the population in each profile. Table 2 displays our initial testing of eight potential model combinations, which involved adjustments in the number of classes and parameterisation (see Appendix D, Table 12). We focused on two specific models for further analysis: the Class Invariant Diagonal Parameterisation (CIDP; Model 1) with five profiles and the Class Invariant Unrestricted Parameterisation (CIRP; Model 3) with five profiles. These models were further examined because they exhibited lower AIC and BIC values.

**Table 2:** Initial Model Testing.

Model	Classes	AIC	BIC	AWE	CLC	KIC	Warnings
1	2	8836.830	8906.219	9054.004	8806.435	8855.830	
1	3	8688.450	8783.860	8987.670	8646.050	8713.450	
1	4	8659.179	8780.610	9040.566	8604.655	8690.179	
**1**	**5**	**8495.218**	**8642.671**	**8958.502**	**8428.839**	**8532.218**	
2	2						Warning
2	3						Warning
2	4						Warning
2	5						Warning
3	2	8589.304	8702.061	8942.998	8539.125	8618.304	
3	3	8586.102	8724.880	9022.247	8523.513	8621.102	
3	4	8543.683	8708.483	9061.828	8469.137	8584.683	
**3**	**5**	**8477.206**	**8667.026**	**9077.376**	**8390.677**	**8524.206**	
6	2						Warning
6	3						Warning
6	4						Warning
6	5						Warning

*Note.* This table presents comparisons between various numbers of profiles for four potential combinations of model parameters, including equal/fixed classes and equal/equal covariances. The results are highlighted (bold) to indicate the best model parameterisation based on the best information criterion. The information criteria used are AIC (Akaike Information Criterion), BIC (Bayesian Information Criterion), AWE (approximate Weight of Evidence Criterion), CLC (classification Likelihood Criterion), and KIC (Kullback Information Criterion).

Table 3 displays additional testing to enhance the fit indices for the CIDP model between the five profiles based on the fit indices. Although the CIRP model with five profiles yielded a better AIC value, the CIDP with five profiles was found to achieve a higher level of classification accuracy (entropy = 0.79), also showing a significant BLRT-p value. As a result, the CIDP model with five profiles was selected due to its optimal fit among the models tested.

**Table 3:** Fit Indices of CIPD With Five Profiles.

Model	Classes	AIC	BIC	Entropy	N_min	BLRT
1	2	8804	8873	**0.799**	0.324	0.01
1	3	8734	8829	0.789	0.216	0.01
1	4	8654	8775	0.728	0.193	0.01
1	5	**8493**	**8640**	0.796	0.119	0.01

*Note.* The table indicates that the CIDP model with five latent profiles exhibits lower AIC and BIC values, and a high entropy value, resulting in improved differentiation between profiles. The information criteria used are AIC (Akaike Information Criterion) and BIC (Bayesian Information Criterion). The BLRT-p (Bootstrapped Likelihood Ratio Test) was employed for comparison. Values highlighted in bold indicate the optimal model based on criteria used.

The observed entropy for the CIDP model surpassed the cut-off point of 0.76 (Larose et al., 2016), indicating an accurate classification of the CIDP five profile structure with over 90 % correctness. The proportion of participants in each estimated profile were *n* = 79 (14.0 %) for Profile 1, *n* = 67 (11.9 %) for Profile 2, *n* = 99 (17.5 %) for Profile 3, *n* = 113 (20.0 %) for Profile 4, and *n* = 207 (36.6 %) for Profile 5 (as seen in Table 4). At this point, it should be noted that the smallest class proportion and number exceed 5 % and/or N > 50 recommended (O’Donnell et al., 2017). Table 4 presents the profiles' standardised mean scores, raw mean scores, and standard deviation for each flow profile.

**Table 4:** Description of Flow Profiles, Including Raw and Standardised Mean Scores of Flow.

Profile	FlowQ1	ZFlowQ1	FlowQ2	ZFlowQ2	FlowQ3	ZFlowQ3	FlowQ4	ZFlowQ4	FlowQ5	ZFlowQ5
1	3.9	0.7	4.9	0.8	4.8	0.6	4.6	0.7	4.0	1.5
2	2.3	−0.4	1.9	− 1.4	2.2	−1.9	2.4	−1.1	2.0	−0.1
3	2.9	0.0	4.2	0.3	3.3	−0.8	2.8	−0.8	2.3	0.1
4	2.3	−0.4	2.1	−1.2	4.5	0.4	3.7	−0.0	1.8	−0.3
5	3.0	0.1	4.7	0.7	4.8	0.6	4.3	0.5	1.6	−0.4

*Note.* Z scores represent standardised scores.

**Fig. 1:**
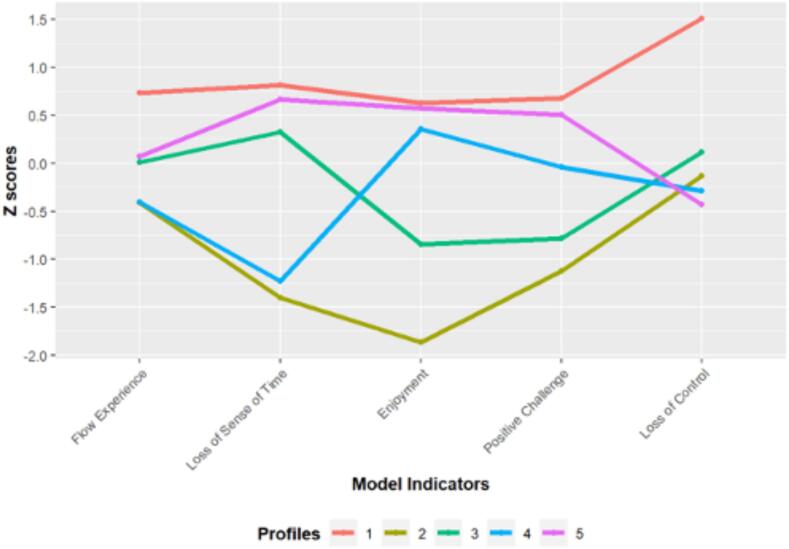
*Flow Latent Profiles. Note.* The plot illustrates five distinct latent profiles considering participants' symptoms of flow measured in standard deviation from the mean, including flow experience, loss of sense of time, employment, positive challenge, and loss of control. The high lines represent high-flow symptoms, the middle lines represent medium-flow symptoms, and the lower lines represent low-flow symptoms.

The latent flow profiles were analysed using raw and standardised reported symptoms to investigate their differing characteristics while maintaining an objective understanding through normal distributions of online flow (RQ1). The findings have revealed that the five latent flow profiles exhibited variations in raw scores and mean values of flow experience, loss of sense of time, enjoyment, positive challenge, and loss of control. Fig. 1 demonstrates mean standardised differences in online flow symptoms across the latent profiles revealed.

Individuals classified in Profile 1 scored in the “High” range for Flow Experience (3.9), and Loss of Control (4.0), scoring above mean flow values for our sample (+0.70SD to + 1.50SD). Consequently, *Profile 1* was defined as “High Flow with High Loss of Control” (HF-HLOC), distinguished by the highest flow symptoms. Participants in *Profile 2* scored in the “Low” range for Flow Experience (2.3), “Low” range for Enjoyment (2.2), remaining below mean flow levels (−1.90SD to − 0.10SD). Thus, *Profile 2* was labelled “Low Flow with Low Enjoyment” (LF-LE). Participants in *Profile 3* scored in the “Average” range for Flow Experience (0), the “Low” range for Enjoyment (3.3) and scored average mean flow levels (−0.80SD to + 0.30SD). This profile was thus defined as “Average Flow with Low Enjoyment” (AF-LE), distinguished by the least deviations in symptom experiences when compared with the other flow profiles. Additionally, participants in *Profile 4* scored in the “Low” range for Flow Experience (2.3), and “High” range for Enjoyment (4.5), scoring average mean flow levels (−1.20SD to + 0.40SD). Hence, this profile was defined as “Low Flow with High Enjoyment” (LF-HE). Finally, participants in *Profile 5* scored in the “High” range for Loss of Sense of Time (4.7), and “Low” range for Loss of Control (1.6), scored above mean flow levels (−0.40SD to + 0.70SD). As a result, *Profile 5* was defined as “High Loss of Time with Low Loss of Control” (HLOT-LLOC).

### 3.2 Hypothesis 1: Flow profiles and IGD

Four one-way ANOVAs were conducted to investigate the differences between IGD and GD scores within each of the five profiles in wave 1 and wave 2. The ANOVA findings for IGD in wave 1 revealed a substantial and statistically significant impact on IGD scores among the distinct profiles, *F*(1, 558) = 21.97, *p* < 0.001. Post hoc pairwise comparisons using t-tests with Bonferroni correction indicated that the differences in IGD in wave 1 were highly significant across Profile 1 (HF-HLOC) and each of the other profiles. Specifically, significant differences were observed when comparing Profile 1 with Profile 2 (*p* < 0.001.), Profile 3 (*p* < 0.001.), Profile 4 (*p* < 0.001.), and Profile 5 (*p* < 0.001.). Additionally, there were significant differences between Profile 2 and Profile 3 (*p* < 0.001) and between Profile 3 and Profile 5 (*p* < 0.05). Moreover, the ANOVA findings for IGD in wave 2 indicated that there was a statistically significant difference in IGD scores among the profiles, *F*(1, 288) = 15.22, *p* < 0.001, with the Bonferroni post hoc analyses revealing significant differences between Profile 1 and each profile such as Profile 2 (*p* < 0.001), Profile 3 (*p* < 0.01), Profile 4 (*p* < 0.01), and Profile 5 (*p* < 0.001).

The ANOVA results for the GD scores in wave 1 and the profiles show a statistically significant effect of GD across profiles, *F*(1, 558) = 25.25, *p* < 0.001), with the profiles also being significant in Bonferroni post hoc analyses (Profile 1 and Profile 2, Profile 3, Profile 4, Profile 5; Profile 2 and Profile 3; Profile 3 and Profile 5; and Profile 4 and Profile 5). Additionally, ANOVA findings on GD scores in wave 2 revealed a significant difference across profiles, *F*(1, 288) = 15.30, *p* < 0.001). Subsequent Bonferroni analyses showed differences between Profile 1 and other profiles, *p* < 0.05 (Profile 2, Profile 3, and Profile 4) in wave 2 (see Appendix C). This supports our hypothesis indicating that participants classified across different flow profiles significantly differ regarding their disordered gaming behaviours experienced concurrently (wave 1) and over time (wave 2).

## 4 Discussion

This study was the first to longitudinally (i.e. over six months) investigate online flow profiles in relation to disordered gaming behaviours, as assessed both via the proposed DSM-5 (APA, 2013) and ICD-11 (WHO, 2019) criteria, in a large sample of 565 gamers. Furthermore, the current project employed a well-validated and widely used international flow scale (i.e., OFQ) and implemented 12 advanced statistical models, varying in parameterisations and number of profiles to detect the optimum number of classes described the sample examined (Rosenberg et al., 2019). The findings suggest the occurrence of five distinct flow profiles. These include *Profile 1* “High Flow with High Loss of Control” (HF-HLOC; 14 %), *Profile 2* “Low Flow with Low Enjoyment” (LF-LE; 11.9 %), *Profile 3* “Average Flow with Low Enjoyment” (AF-LE; 17.5 %), *Profile 4* “Low Flow with High Enjoyment (LF-HE; 20.2 %), and *Profile 5* ”High Loss of Sense of Time with Low Loss of Control“ (HLOT-LLOC; 36.5 %). As predicted, participants categorised into distinct flow profiles show significant differences in their concurrent (wave 1) and longitudinal (wave 2) disordered gaming behaviours, irrespective of whether the APA (2013) or the WHO (2019) criteria were employed.

### 4.1 Different flow profiles within the gaming community

The findings suggest five diverse profiles of flow present among this gaming population. The flow levels within each profile exhibited qualitative differences (i.e. not homogeneously differing in intensity across the number of flow criteria employed). Specifically, participants in the HF-HLOC profile showed high flow, loss of sense of time, and loss of control, ranging between 0.70 and 1.50 *SD*s above mean sample scores. Participants in the LF-LE profile showed low flow and low enjoyment, remaining below mean flow levels. Participants in the AF-LE showed average flow, low enjoyment, and remained within average mean flow levels. Additionally, participants in the LF-HE profile showed low flow, and high enjoyment, scoring average mean flow levels. Finally, participants in the HLOT-LLOC profile showed high loss of sense of time, and low loss of control, and ranged within sample mean levels (−0.40SD to + 0.70SD). This suggests that participants in the HF-HLOC profile were at higher risk of experiencing loss of control compared with other latent traits.

These findings both align and diverge from previous literature. While past literature has highlighted the significance of flow in disordered usage (Hu et al., 2019, Khantzian, 1997, Stavropoulos et al., 2018b), identifying distinct flow profiles/typologies adds a novel dimension to the existing literature. The current study also supports previous research demonstrating the heterogeneous nature of the gaming population defined by distinct characteristics (Billieux et al., 2015 l; Colder Carras and Kardefelt-Winther, 2018, Lee et al., 2017, Lemmens et al., 2015; Tullet-Prado et al., 2021). While flow has been examined through various perspectives in research (e.g., Flow State Scale; [Jackson & Marsh, 1996], and multiple factors have been considered as contributors to flow (e.g., 36-item instrument in Jackson & Marsh, [1996]; 5-item instrument in OFQ, [Chen et al., 1999]), this study reveals that the most consistently influential factor in the flow experience through the OFQ is 'loss of sense of time'. As illustrated in Fig. 1, individuals classed in HF-HLOC, AF-LE, and HLOT-LLOC all experience a general state of flow and a high loss of sense of time.

In contrast, LF-LE and LF-HE exhibit low levels of loss of sense of time, consequently experiencing low states of flow. Additionally, AF-LE demonstrates an average state of flow experience, coupled with reduced enjoyment and minimal positive challenge. These findings suggest that the phenomenon of losing the sense of time may serve as the most pivotal facilitator for distinguishing flow states and profiles during gaming. However, the presence of positive challenge and enjoyment as indicators appear to act as potential moderators/barriers to reaching high-flow states. Furthermore, loss of control appeared to have the most substantial deviation from the mean in terms of flow indicators in HF-HLOC. This notable difference in loss of control is plausible, given the logical association between heightened loss of control and high flow states when being profoundly absorbed in a gaming activity (Stavropoulos et al., 2019a). However, 'loss of control' manifested relatively modestly across the other profiles, suggesting that high loss of control might constitute an additional important factor in facilitating higher flow states. This aspect may be worth exploring/emphasising in future flow states/profiles investigations. These results align with past literature suggesting the occurrence of different types/profiles of gamers, which in turn influence their potential to exhibit disordered gaming, with the vast majority of gamers demonstrating healthy/adaptive gaming involvement, highlighting the need not to pathologize gaming as a leisure activity (Billieux et al., 2015 l; Colder Carras and Kardefelt-Winther, 2018, Lee et al., 2017, Lemmens et al., 2015, Tullett-Prado et al., 2021).

### 4.2 Relationship between flow profiles and disordered gaming behaviours

Consistent with previous research, the present study reported a significant association between gamers' flow levels and IGD levels (Stavropoulos et al., 2018b, Stavropoulos et al., 2021), both concurrently (wave 1) and over time (wave 2). Specifically, the findings revealed HF-HLOC to have a significant difference between the other IGD profiles (APA, 2013) and GD (WHO, 2019) in both the first and second waves of data. The aetiological models, which explore the interplay between individual vulnerabilities and immersive game elements driving IGD, further substantiate these findings (Brand et al., 2016, Griffiths et al., 2017, Kardefelt-Winther, 2014). Particularly, the self-medication (Kantzian, 1997) and CIU models (Kardefelt-Winther, 2014) suggest that online gaming can alleviate negative affect among stressed individuals (Kovacs et al., 2022, Griffiths et al., 2017) and serve as an adaptive mechanism for coping with adverse life events through satisfying in-game experiences (Kardefelt-Winther, 2014).

Practically, these findings hold promise for the development of assessment, prevention, and treatment approaches when addressing disordered gaming behaviours. Specifically, observed flow profiles suggest that individuals who experience a high loss of sense of time, along with high enjoyment and high loss of control, tend to have heightened flow experiences and are more susceptible to the development of disordered gaming. Additionally, individuals who exhibit HF-HLOC symptoms should be recognised as at-risk and prioritised for assessment and prevention efforts. This emphasises the need for comprehensive diagnostic and assessment procedures, including those that evaluate loss of control, when assessing flow symptoms in individuals seeking help for disordered gaming behaviours. Similarly, flow experiences should be consistently addressed as central disordered gaming perpetuating/maintaining factors in treatment case formulations for disordered gaming cases, while ways to introduce positive flow experiences (e.g. experiencing flow though feasible educational and/or employment progression) outside the game should also be considered.

### 4.3 Limitations and future research

Despite the novel contributions of this study to the extant literature, it is important to consider its potential limitations. Firstly, the study is subject to selection bias due to its reliance on non-random community sampling, which restricts the wide-scale generalisation of the findings (Emerson, 2021). To mitigate these biases, future research should employ random stratified sampling to obtain a more representative sample of the population, and/or target clinical samples, thereby enhancing the applicability and generalisability of the findings to the broader population and/or diagnosed groups. Secondly, the use of self-report questionnaires in this study may be affected by social desirability bias, whereby participants may have adjusted their responses in a more positive direction to conform with societal norms and expectations. Thirdly, our study's second wave comprised a smaller number of adolescent participants, which limits our knowledge of flow typologies concerning disordered gaming within this age group. Future studies would, therefore, benefit by including a more extensive adolescent sample when further examining flow profiles in disordered gaming, targeting diagnosed disordered gaming populations and using multi-method designs integrating both self-report and objective measures such as game-time monitoring applications.

## 5 Conclusion

In light of such limitations, these findings stipulate significant future research directions. Future researchers may wish to conduct longitudinal studies to track changes in flow profiles over time and their impact on disordered gaming development. Furthermore, prospective studies could focus on enhancing game challenges to help individuals allocated in profiles that are characterised by limited positive challenge and enjoyment, thereby helping them attain an adequate level of online flow during their gaming experience to enhance enjoyment. Moreover, examining the efficacy of psychological interventions aimed at mitigating the 'loss of control’ flow feature/item could yield valuable insights for refining preventative and treatment measures to address individuals presenting with high disordered gaming risk. Future researchers should additionally consider exploring the potential of network analysis to establish a unified way of understanding flow and confirm the identification of the central behaviours that are associated with in-game flow experiences. This avenue may hold promise for intriguing insights in the literature.

## 6 Author Agreement Statement

We the undersigned declare that this manuscript is original, has not been published before and is not currently being considered for publication elsewhere.

We confirm that the manuscript has been read and approved by all named authors and that there are no other persons who satisfied the criteria for authorship but are not listed. We further confirm that the order of authors listed in the manuscript has been approved by all of us.

We understand that the Corresponding Author is the sole contact for the Editorial process. He is responsible for communicating with the other authors about progress, submissions of revisions and final approval of proofs.

Signed by all authors as follows: Trent Footitt, Natasha Christofi, Dylan R Poulus, Michelle Colder Carras, and Vasileios Stavropoulos.

## CRediT authorship contribution statement

**Trent Footitt:** Writing – original draft, Methodology, Investigation, Formal analysis, Data curation, Conceptualization. **Natasha Christofi:** Writing – original draft, Methodology, Formal analysis. **Dylan R Poulus:** Writing – review & editing, Visualization, Methodology, Formal analysis. **Michelle Colder Carras:** Writing – review & editing, Supervision, Data curation. **Vasileios Stavropoulos:** Writing – review & editing, Supervision, Resources, Methodology, Investigation, Formal analysis, Data curation, Conceptualization.

## Declaration of competing interest

The authors declare that they have no known competing financial interests or personal relationships that could have appeared to influence the work reported in this paper.

## Figures and Tables

**Table 1a:** Gender-Based Attrition in First and Second Waves.

				Gender															
Attrition W1W2				Man (cisgender)		Woman (cisgender)		Man (transgender)		Woman (transgender)		Non binary		Not Listed		Prefer not to say		Total	
First and second wave		Observed		139		135		3		1		8		2		1		289	
		% within column		49.1 %		52.1 %		75.0 %		100.0 %		66.7 %		66.7 %		33.3 %		51.2 %	
Only first wave		Observed		144		124		1		0		4		1		2		276	
		% within column		50.9 %		47.9 %		25.0 %		0.0 %		33.3 %		33.3 %		66.7 %		48.8 %	
Total		Observed		283		259		4		1		12		3		3		565	
		% within column		100.0 %		100.0 %		100.0 %		100.0 %		100.0 %		100.0 %		100.0 %		100.0 %	

**Table 1b:** Chi-Square Analyses Regarding Gender-Related Attrition

		Value		df		p	
χ^2^		4.26		6		0.642	
N		565					

*Note.* This Chi-Square test indicates the significance of attrition regarding gender in first and second waves.

**Table 1c:** Effect Size of Gender-Based Attrition in First and Second Waves.

		Value	
Phi-coefficient		NaN	
Cramer's V		0.0868	

*Note.* This table demonstrates the strength and significance of association between gender-based attrition in the first and second waves.

**Table 2a:** Attrition Regarding Sexual Orientation in First and Second Waves

		SexOrient_W1					
AttritionW1W2		Heterosexual -Straight	Homosexual	Bisexual	Asexual	Other (Define)	Total
First and second wave	Observed	201	23	37	5	5	271
	% of total	41.2 %	4.7 %	7.6 %	1.0 %	1.0 %	55.5 %
Only first wave	Observed	158	13	38	0	8	217
	% of total	32.4 %	2.7 %	7.8 %	0.0 %	1.6 %	44.5 %
Total	Observed	359	36	75	5	13	488
	% of total	73.6 %	7.4 %	15.4 %	1.0 %	2.7 %	100.0 %

*Note.* This table demonstrates the proportion of participant dropout based on sexual orientation between the first and second waves. The question was asked only to adult participants as per ethics approvals received.

**Table 2b:** Chi-Square Analyses Examining Attrition Based on Sexual Orientation

		Value		df		p	
χ^2^		7.75		4		0.101	
N		488					

*Note.* This Chi-Square test indicates the significance of attrition regarding sexual orientation in first and second waves. The question was asked only to adult participants as per ethics approvals received.

**Table 2c:** Effect Size of Sexual Orientation-Based Attrition in First and Second Waves.

		Value	
Phi-coefficient		NaN	
Cramer's V		0.126	

*Note.* This table demonstrates the strength and significance of association between attrition and sexual orientation in the first and second waves. The question was asked only to adult participants as per ethics approvals received.

**Table 3a:** Attrition Regarding Ancestry in First and Second Waves

				Background											
AttritionW1W2				Aus./Engl.		Chinese		German		Indian		Other		Total	
First and second wave		Observed		202		10		7		7		63		289	
		% within column		49.3 %		50.0 %		100.0 %		70.0 %		53.4 %		51.2 %	
Only first wave		Observed		208		10		0		3		55		276	
		% within column		50.7 %		50.0 %		0.0 %		30.0 %		46.6 %		48.8 %	
Total		Observed		410		20		7		10		118		565	
		% within column		100.0 %		100.0 %		100.0 %		100.0 %		100.0 %		100.0 %	

*Note.* This table illustrates the proportion of participant dropout based on background between the first and second waves.

**Table 3b:** Chi-Square Analyses Examining Attrition Based on Ancestry

		Value		df		p	
χ^2^		8.94		4		0.063	
N		565					

*Note.* This Chi-Square test indicates the significance of attrition regarding participant background in first and second waves.

**Table 3c:** Effect Size of Attrition Based on Ancestry

		Value
Phi-coefficient		NaN
Cramer's V		0.126

*Note.* This table demonstrates the strength and significance of association between attrition and ancestry in the first and second waves.

**Table 4a:** Attrition Regarding Occupational Status in First and Second Waves.

		OccupationalStatus_w1							
AttritionW1W2		Full-Time Employed	Part-Time Employed	Student	Trainee	Not Currently Working	On Temporary Leave	Other (Define)	Total
First and second wave	Observed	159	43	30	1	18	4	16	271
	% of total	32.4 %	8.8 %	6.1 %	0.2 %	3.7 %	0.8 %	3.3 %	55.3 %
Only first wave	Observed	112	34	34	1	14	1	23	219
	% of total	22.9 %	6.9 %	6.9 %	0.2 %	2.9 %	0.2 %	4.7 %	44.7 %
Total	Observed	271	77	64	2	32	5	39	490
	% of total	55.3 %	15.7 %	13.1 %	0.4 %	6.5 %	1.0 %	8.0 %	100.0 %

*Note.* On temporary leave includes Education Leave, Public Service Leave, Training, Maternity Leave.

**Table 4b:** Chi-Square Analyses Examining Attrition Based on Occupational Status

		Value		df		p
χ^2^		7.58		6		0.271
N		490				

*Note.* This Chi-Square test indicates the significance of attrition regarding occupational status in first and second waves.

**Table 4c:** Effect Size of Attrition Based on Occupational Status.

		Value	
Phi-coefficient		NaN	
Cramer's V		0.124	

*Note*. This table demonstrates the strength and significance of attrition based on occupational status in the first and second waves.

**Table 5a:** Attrition Regarding Highest Level of Completed Education in First and Second Waves

		HighestLevelofCompletedEducation_w1								
AttritionW1W2		Professional Degree (i.e. MD, JD etc completed)	PhD Degree (Completed)	Postgraduate Studies (MSc Completed)	Undergraduate University Course (completed)	Intermediate between secondary level and university (e.g. Technical training)	Senior secondary school (Years 11 to 12)	Secondary school (Years 7 to 10)	Other (define)	Total
First and second wave	Observed	8	12	38	104	50	47	3	8	270
	% of total	1.6 %	2.5 %	7.8 %	21.3 %	10.2 %	9.6 %	0.6 %	1.6 %	55.2 %
Only first wave	Observed	2	5	29	72	47	54	6	4	219
	% of total	0.4 %	1.0 %	5.9 %	14.7 %	9.6 %	11.0 %	1.2 %	0.8 %	44.8 %
Total	Observed	10	17	67	176	97	101	9	12	489
	% of total	2.0 %	3.5 %	13.7 %	36.0 %	19.8 %	20.7 %	1.8 %	2.5 %	100.0 %

**Table 5b:** Chi-Square Analyses Examining Attrition Based on Highest Level of Completed Education

		Value		df		p
χ^2^		11.2		7		0.129
N		489				

*Note.* This Chi-Square test indicates the significance of attrition regarding the highest level of completed education in first and second waves.

**Table 5c:** Effect Size of Attrition Based on Highest Level of Completed Education

		Value	
Phi-coefficient		NaN	
Cramer's V		0.152	

*Note.* This table demonstrates the strength and significance of attrition based on the highest level of education completed in the first and second waves.

**Table 6a:** Attrition Regarding Relationship Status in First and Second Waves

		RelationshipStatus_w1					
Attrition W1W2		Single	In a romantic relationship	Engaged	Married	De facto	Total
First and second wave	Observed	77	82	13	89	10	271
	% of total	15.7 %	16.7 %	2.7 %	18.2 %	2.0 %	55.3 %
Only first wave	Observed	71	75	11	56	6	219
	% of total	14.5 %	15.3 %	2.2 %	11.4 %	1.2 %	44.7 %
Total	Observed	148	157	24	145	16	490
	% of total	30.2 %	32.0 %	4.9 %	29.6 %	3.3 %	100.0 %

*Note.* A romantic relationship is defined as a romantic commitment of intensity between two individuals of the same or the opposite sex (When you like a guy [girl] and he [she] likes you back).

**Table 6b:** Chi-Square Analyses Examining Attrition Based on Relationship Status

		Value		df		p	
χ^2^		3.76		4		0.440	
N		490					

*Note.* This Chi-Square test indicates the significance of attrition regarding relationship status in the first and second waves.

**Table 6c:** Effect Size of Attrition Based on Relationship Status

		Value	
Phi-coefficient		NaN	
Cramer's V		0.0876	

*Note.* This table demonstrates the strength and significance of attrition based on relationship status in the first and second waves.

**Table 7:** Comparisons and Effect Size of Attrition Based on Age, Gaming & Social Media Usage Years and Daily Time Consumed During the Week and the Weekend.

				Statistic		df		p		Mean difference		SE difference				Cohen's d /Effect Size	
Gaming years		Student's t		-3.466^a^		554		< .001		-1.3068		0.377				-0.2942	
		Welch's t		-3.509		526		< .001		-1.3068		0.372				-0.2960	
Mean daily gaming time in the week		Student's t		-0.873		555		0.383		-0.1345		0.154				-0.0741	
		Welch's t		-0.870		539		0.385		-0.1345		0.155				-0.0739	
Mean daily gaming time in the weekend		Student's t		-0.159		553		0.874		-0.0324		0.204				-0.0135	
		Welch's t		-0.159		550		0.874		-0.0324		0.204				-0.0135	
Age		Student's t		4.967		560		< .001		4.3653		0.879				0.4192	
		Welch's t		4.959		553		< .001		4.3653		0.880				0.4188	
Social Media usage years		Student's t		2.501		556		0.013		0.9300		0.372				0.2118	
		Welch's t		2.502		556		0.013		0.9300		0.372				0.2118	
Mean daily social media time in the week		Student's t		-2.313		543		0.021		-0.4273		0.185				-0.1983	
		Welch's t		-2.309		535		0.021		-0.4273		0.185				-0.1982	
Mean daily social media time in the weekend		Student's t		-2.468	^a^	541		0.014		-0.5233		0.212				-0.2120	
		Welch's t		-2.447		501		0.015		-0.5233		0.214				-0.2111	

^a^Levene's test is significant (p < .05), suggesting a violation of the assumption of equal variances.

**Table 8a:** Gaming Disorder Test wave 1 reliability analysis.

		mean		SD		Cronbach's α		McDonald's ω	
GDT	_W1	2.18		0.658		0.808		0.812	

**Table 8b:** Gaming Disorder Wave 1 reliability analysis, if items deleted.

								if item dropped			
		mean		SD		item-rest correlation		Cronbach's α		McDonald's ω	
GD_Q1_W1		2.26		0.797		0.657		0.745		0.755	
GD_Q2_W1		2.41		0.844		0.621		0.761		0.769	
GD_Q3_W1		2.23		0.948		0.674		0.738		0.744	
GD_Q4_W1		1.79		0.694		0.568		0.788		0.791	

**Table 8c:** Gaming Disorder Wave 2 reliability analysis

		mean		SD		Cronbach's α	McDonald's ω		
GD_W2		1.89		0.756		0.854		0.862	

**Table 8d:** Gaming Disorder Test Wave 2 reliability analysis, if items deleted

								if item dropped			
		mean		SD		item-rest correlation		Cronbach's α		McDonald's ω	
GD_Q1_W2		2.00		0.913		0.753		0.790		0.809	
GD_Q2_W2		2.19		0.984		0.697		0.816		0.831	
GD_Q3_W2		2.01		1.027		0.755		0.791		0.806	
GD_Q4_W2		1.38		0.656		0.631		0.850		0.852	


**Table 9a:** Internet Gaming Disorder Wave 1 reliability analysis

		mean		SD		Cronbach's α		McDonald's ω	
IGD9	_W1	1.91		0.636		0.846		0.858	

**Table 9b:** Internet Gaming Disorder Wave 1 reliability analysis, if items deleted

								if item dropped			
		mean		SD		item-rest correlation		Cronbach's α		McDonald's ω	
IGD9_Q1_W1		2.37		1.049		0.588		0.827		0.844	
IGD9_Q2_W1		1.84		0.921		0.717		0.814		0.829	
IGD9_Q3_W1		1.91		0.990		0.662		0.819		0.837	
IGD9_Q4_W1		1.69		0.890		0.711		0.815		0.828	
IGD9_Q5_W1		2.09		1.068		0.605		0.825		0.841	
IGD9_Q6_W1		1.63		0.894		0.600		0.826		0.839	
IGD9_Q7_W1		1.41		0.749		0.464		0.840		0.852	
IGD9_Q8_W1		2.95		1.221		0.366		0.858		0.863	
IGD9_Q9_W1		1.30		0.645		0.448		0.841		0.853	

**Table 9c:** Internet Gaming Disorder Wave 2 reliability analysis

		mean		SD		Cronbach's α		McDonald's ω	
IGD9	_W2	1.82		0.644		0.861		0.871	

**Table 9d:** Internet Gaming Disorder Scale Wave 2 reliability analysis, if items deleted

								if item dropped			
		mean		SD		item-rest correlation		Cronbach's α		McDonald's ω	
IGD9_Q1_W2		2.26		1.055		0.623		0.843		0.857	
IGD9_Q2_W2		1.76		0.914		0.715		0.834		0.846	
IGD9_Q3_W2		1.89		0.996		0.716		0.833		0.848	
IGD9_Q4_W2		1.63		0.903		0.724		0.834		0.845	
IGD9_Q5_W2		1.93		1.045		0.637		0.842		0.856	
IGD9_Q6_W2		1.49		0.824		0.617		0.845		0.855	
IGD9_Q7_W2		1.28		0.697		0.507		0.855		0.864	
IGD9_Q8_W2		2.92		1.221		0.435		0.869		0.872	
IGD9_Q9_W2		1.25		0.612		0.419		0.861		0.871	

**Table 10a:** Flow Wave 1 reliability analysis

		mean		SD		Cronbach's α		McDonald's ω	
Flow	_W1	3.36		0.799		0.659		0.680	

**Table 10b:** Online Flow Wave 1 reliability analysis, if items deleted

								if item dropped			
		mean		SD		item-rest correlation		Cronbach's α		McDonald's ω	
FlowQ1_W1		2.82		1.337		0.403		0.613		0.659	
FlowQ2_W1		3.81		1.359		0.464		0.582		0.623	
FlowQ3_W1		4.25		0.977		0.472		0.591		0.594	
FlowQ4_W1		3.81		1.184		0.483		0.575		0.585	
FlowQ5_W1		2.12		1.247		0.276		0.669		0.696	

**Table 10c:** Online Flow Wave 2 reliability analysis.

		mean		SD		Cronbach's α		McDonald's ω	
Flow	_W2	3.28		0.781		0.670		0.690	

**Table 10d:** Online Flow Wave 2 reliability analysis, if items deleted.

								if item dropped			
		mean		SD		item-rest correlation		Cronbach's α		McDonald's ω	
FlowQ1_W2		2.63		1.306		0.408		0.628		0.670	
FlowQ2_W2		3.81		1.249		0.550		0.557		0.607	
FlowQ3_W2		4.25		0.923		0.490		0.603		0.617	
FlowQ4_W2		3.73		1.206		0.430		0.616		0.645	
FlowQ5_W2		1.97		1.225		0.282		0.683		0.705	

**Figure 2.1:**
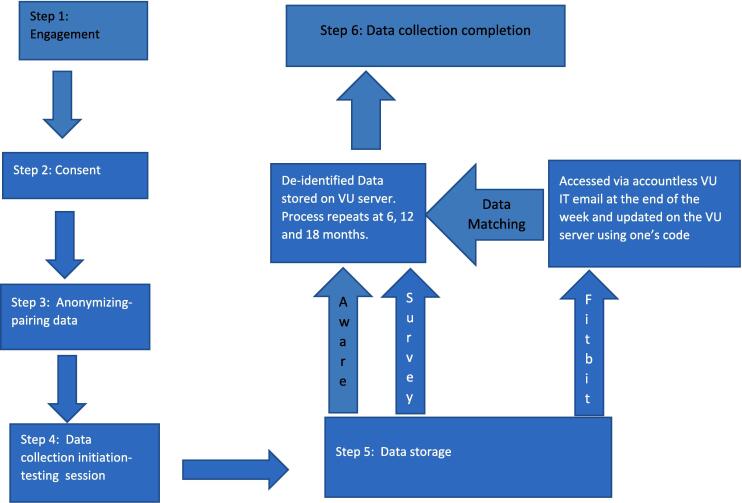


**Figure 2.2:**
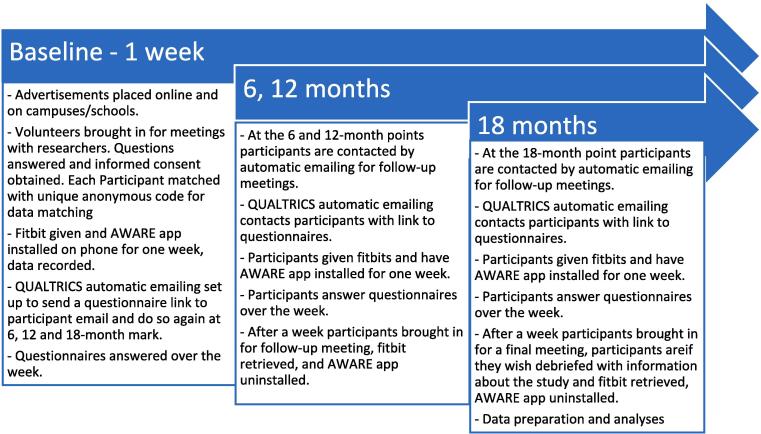


**Fig. 3.1:**
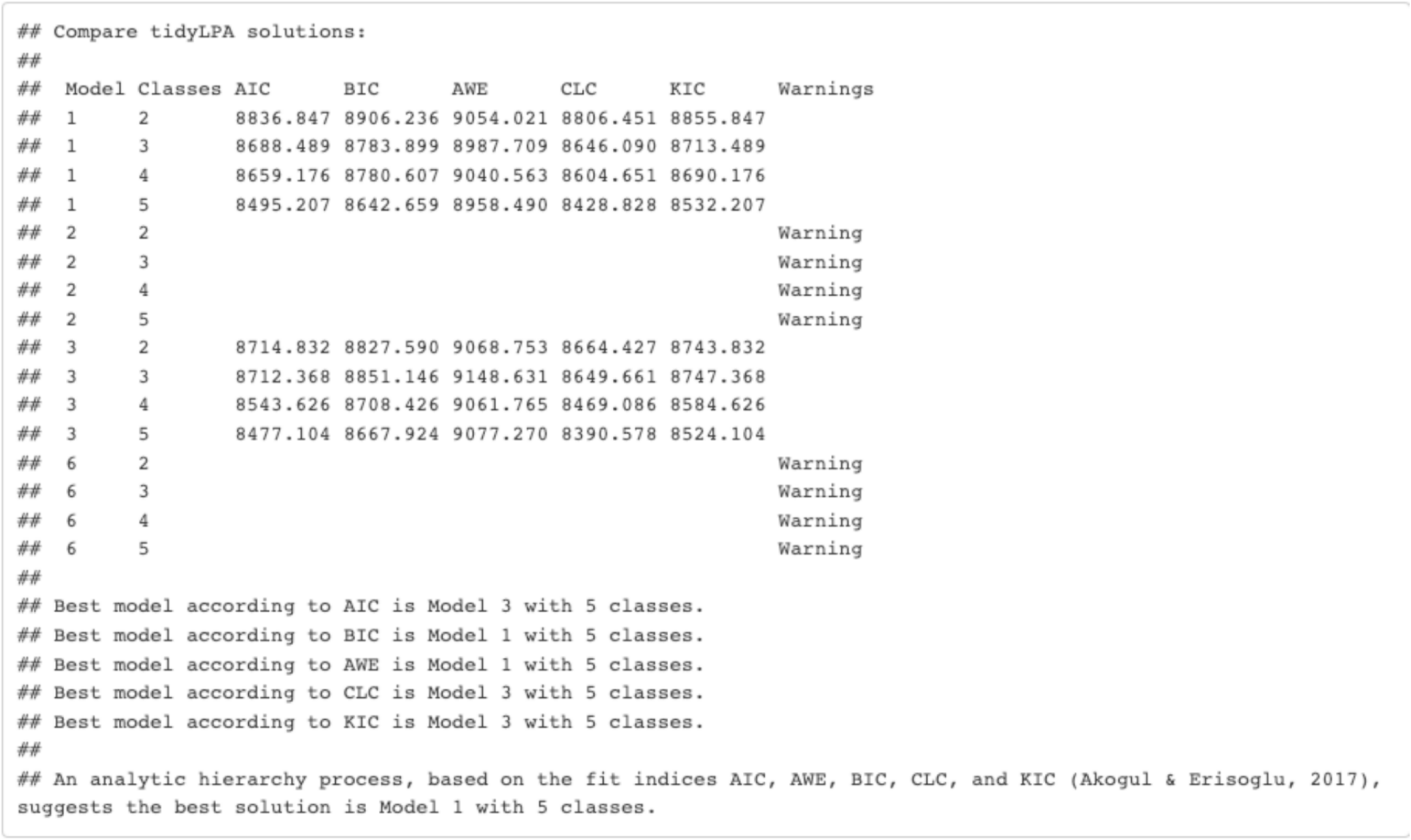
Initial Model Testing19051309769.

**Fig. 3.2:**
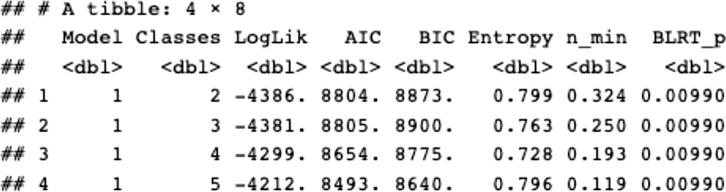
Fit Indices for Selecting the Best Model of CIUP Profiles.

**Fig. 4.1:**
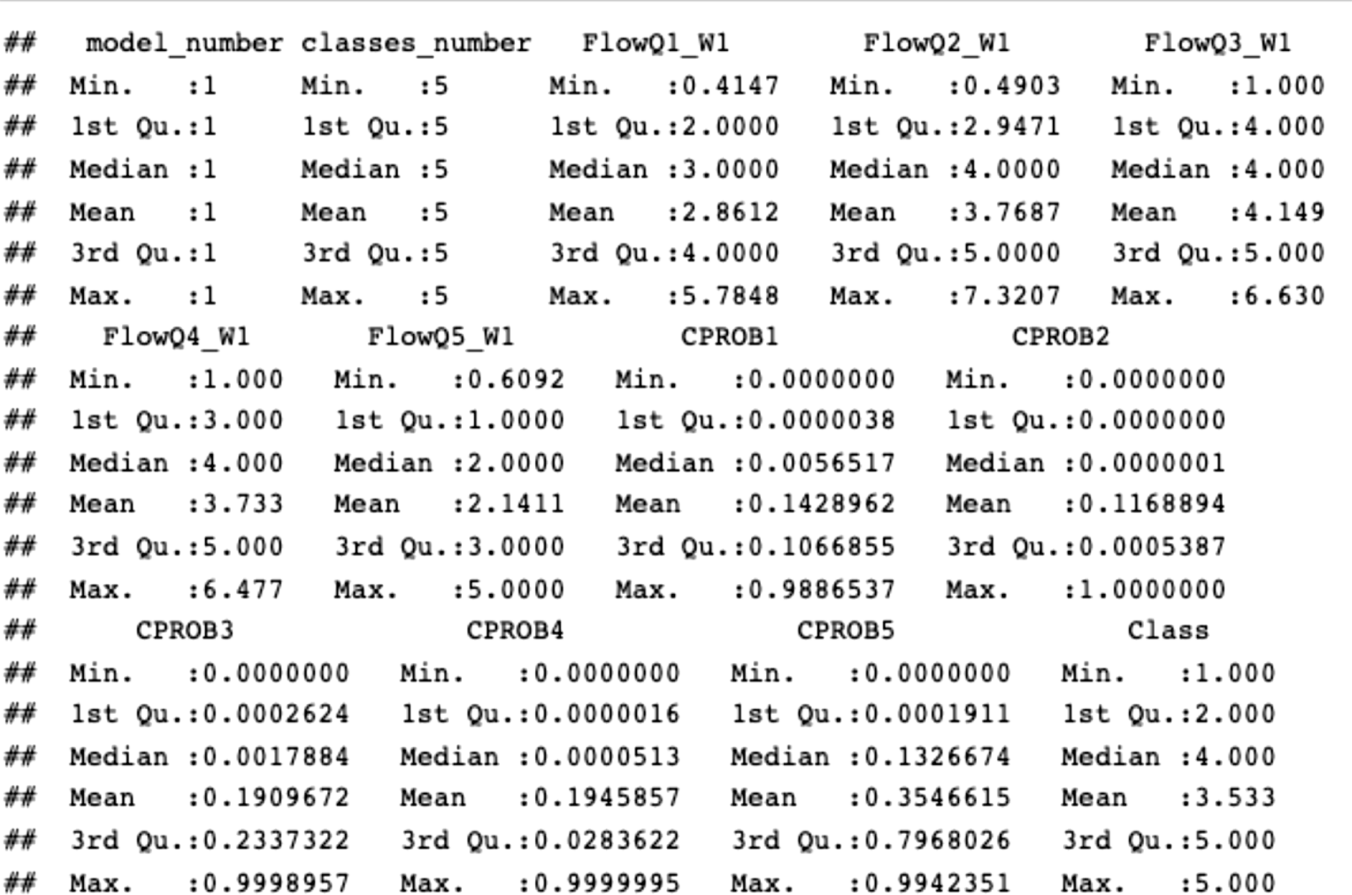
Proportion of participants in each profile.

**Fig. 4.2:**
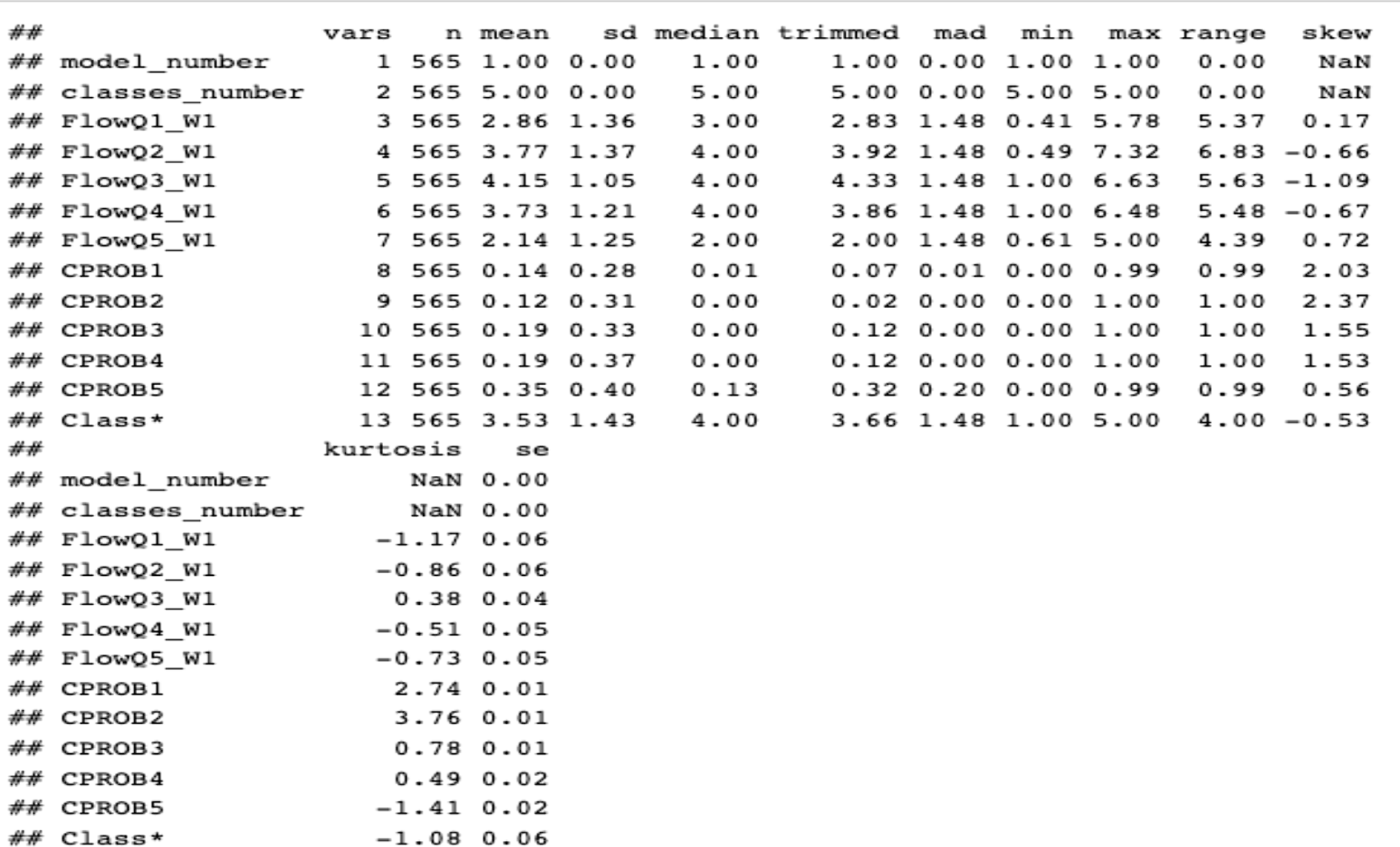
**D**escription of Flow Profiles Including Raw Scores of Flow.

**Fig. 4.3:**
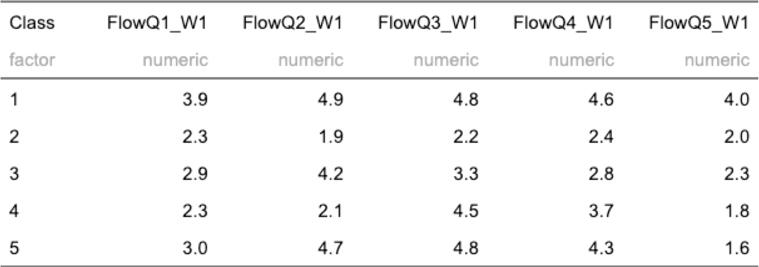
Raw Scores of Flow in Wave 1.

**Fig. 4.4:**
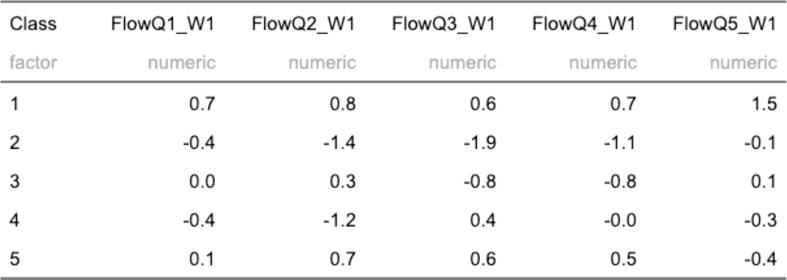
Standardised Mean Values of Flow in Wave 119051372982.

**Fig. 5.1:**
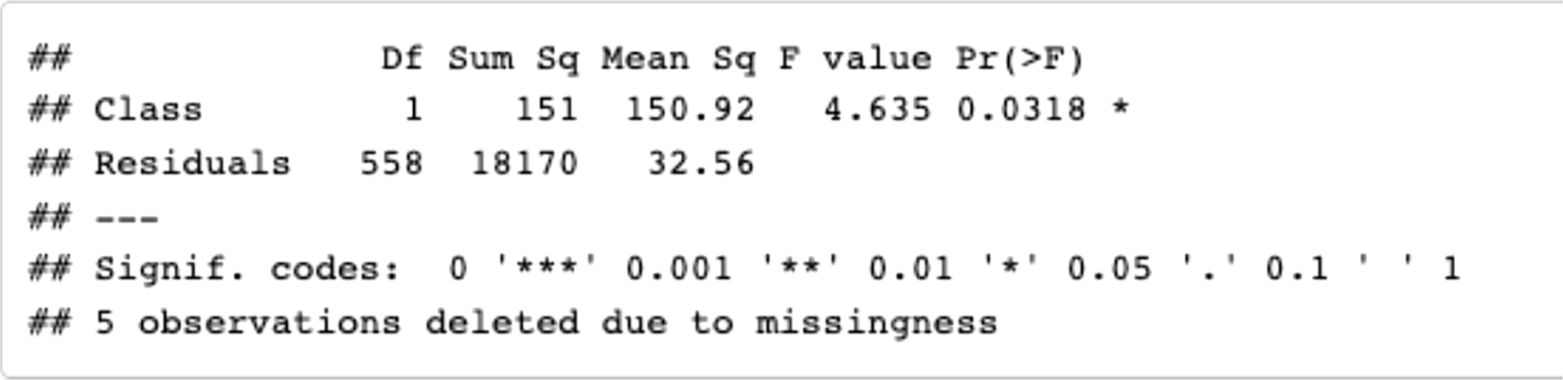
One Way Anova Between Flow and IGDS9-SF in Wave 119051371475.

**Fig. 5.2:**
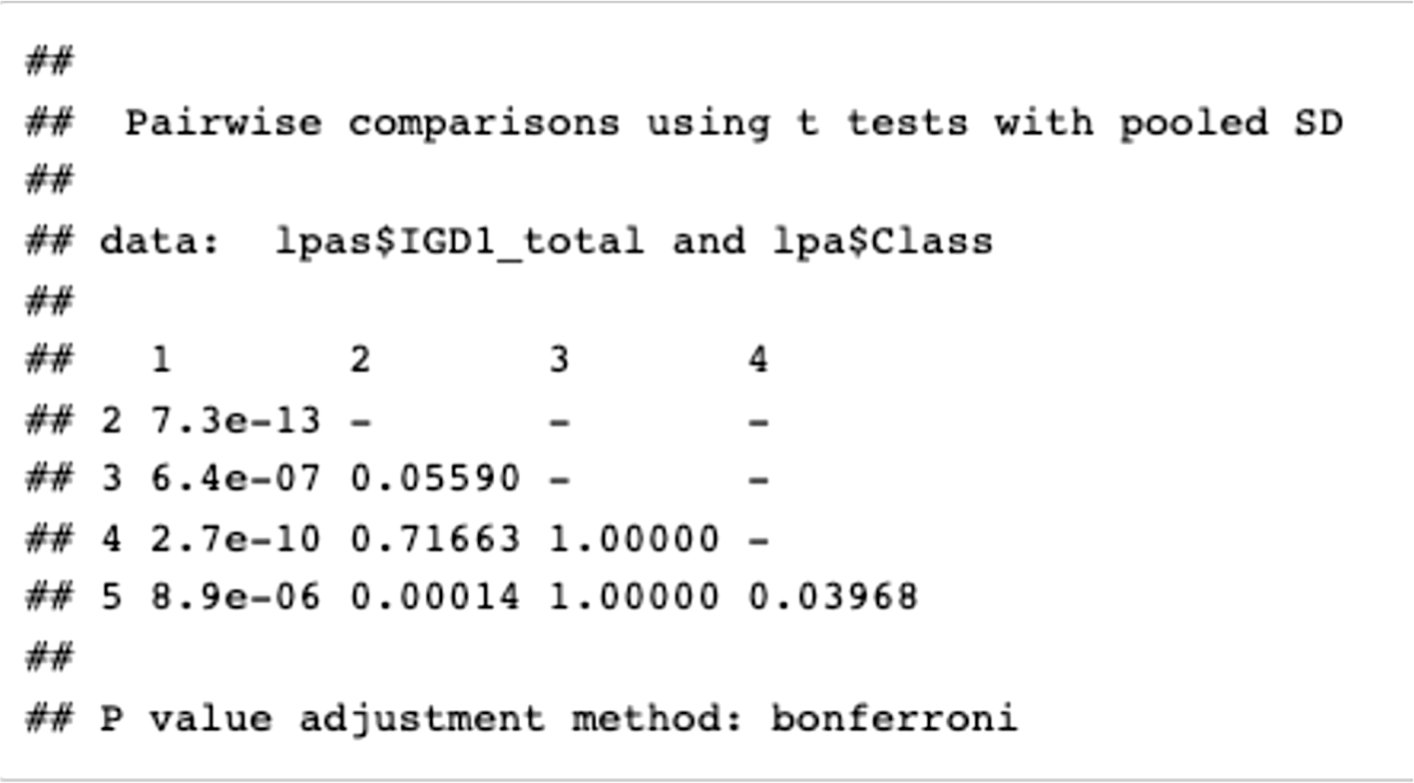
Post Hoc Bonferroni Pairwise T-test IGDS9-SF Comparisons in Wave 1.

**Fig. 5.3:**
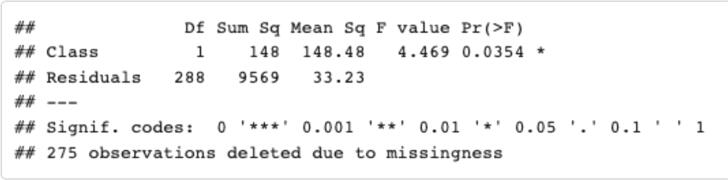
One Way Anova Between Flow and IGDS9-SF in Wave 2.

**Fig. 5.4:**
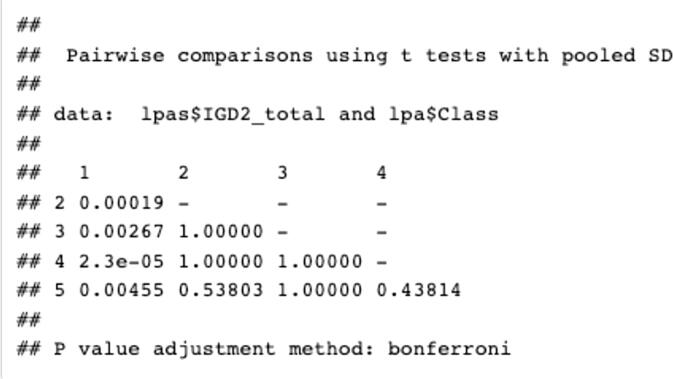
Post Hoc Bonferroni Pairwise T-test IGDS9-SF Comparisons in Wave 2.

**Fig. 5.5:**
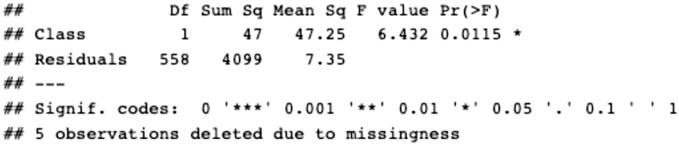
One Way Anova Between Flow and GDT in Wave 1.

**Fig. 5.6:**
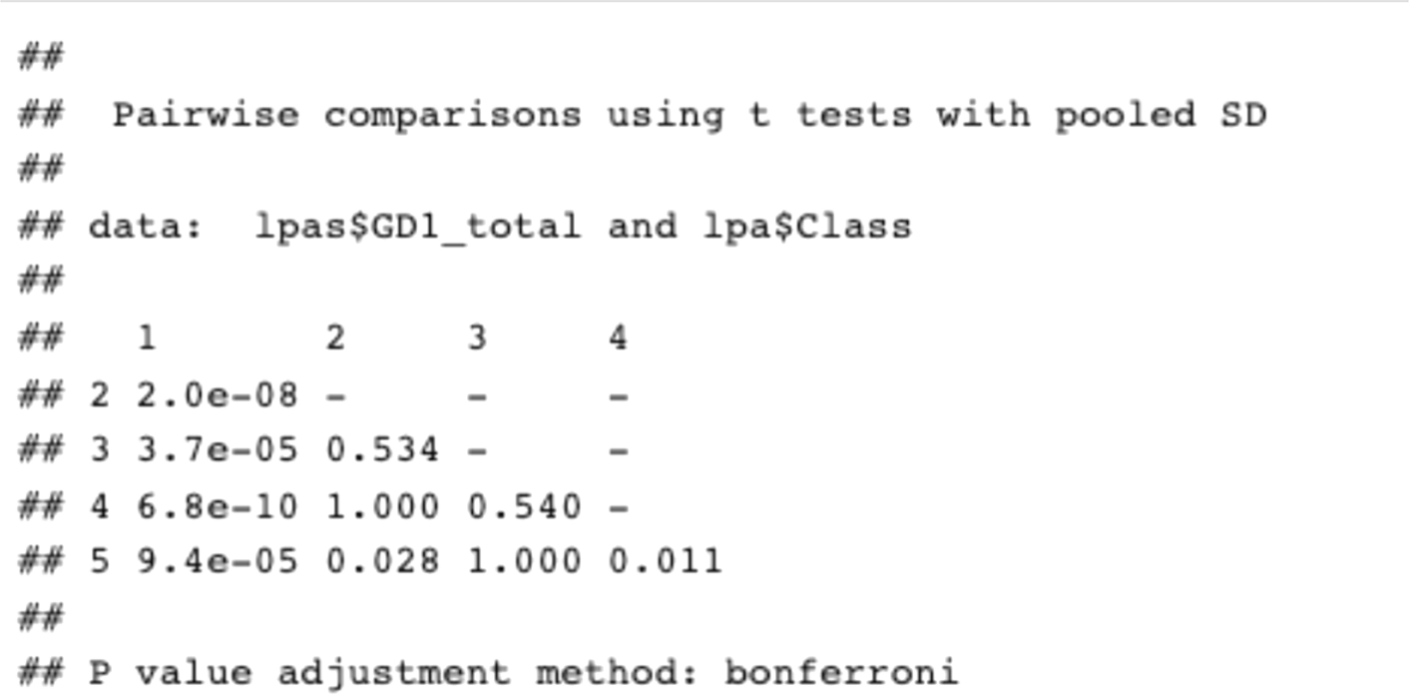
Post Hoc Bonferroni Pairwise T-test GDT Comparisons in Wave 1.

**Fig. 5.7:**
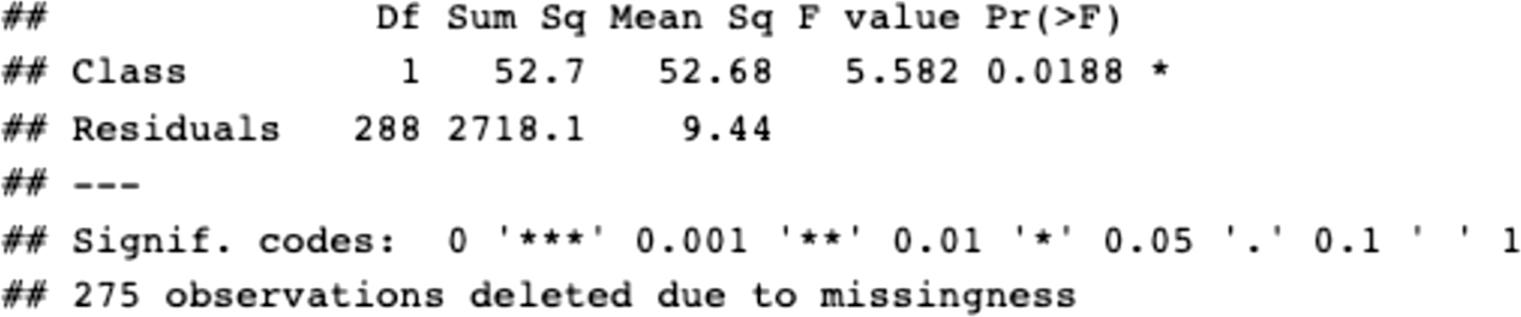
One Way Anova Between Flow and GDT in Wave 2.

**Fig. 5.8:**
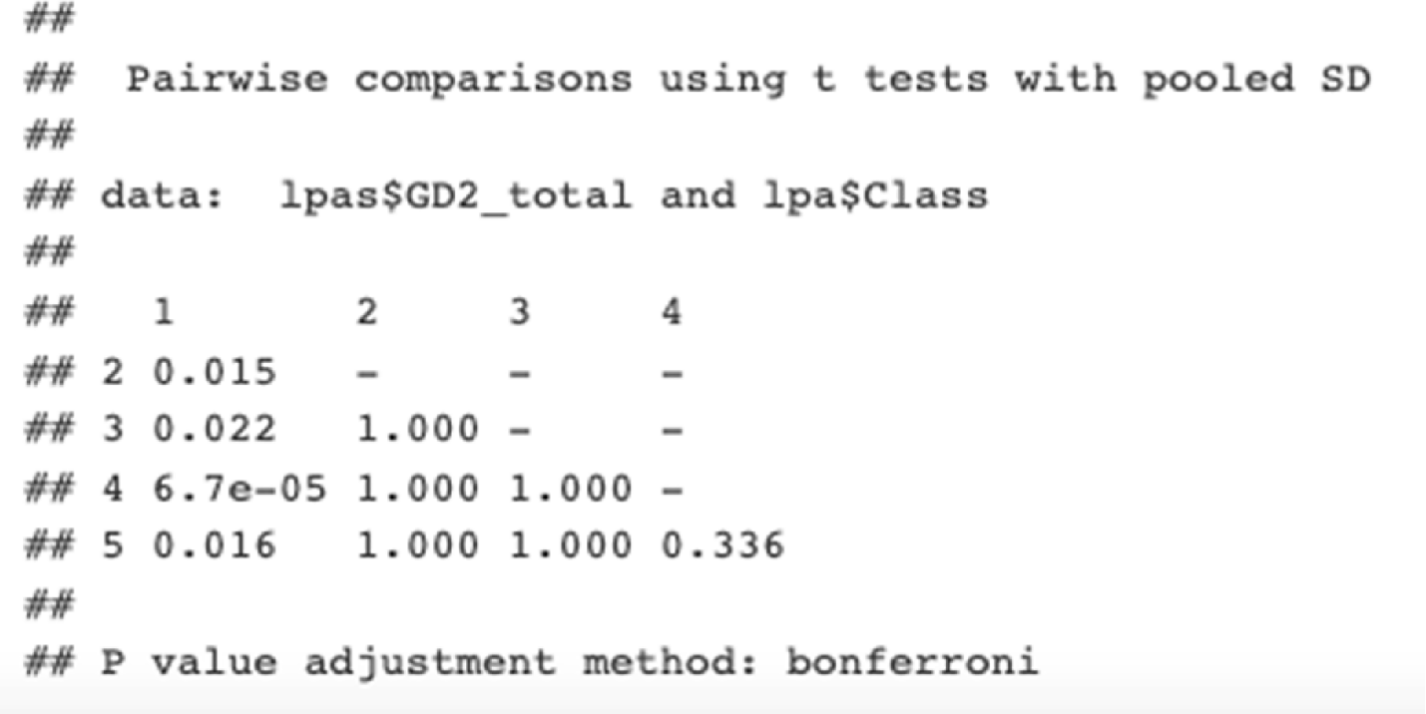
Post Hoc Bonferroni Pairwise T-test GDT Comparisons in Wave 219051372982.

**Table 11:** Participants' sociodemographic, gaming and social media usage information at time point 1.

		Count	Total	Proportion
Gender	Man (cisgender)	283	565	0.501
	Woman (cisgender)	259	565	0.458
	Man (transgender)	4	565	0.007
	Woman (transgender)	1	565	0.002
	Nonbinary	12	565	0.021
	Not Listed (Feel free to write your gender below)	3	565	0.005
	Prefer not to say	3	565	0.005
Sexual Orientation	Heterosexual-Straight	202	272	0.743
	Homosexual	23	272	0.085
	Bisexual	37	272	0.136
	Asexual	5	272	0.018
	Other	5	272	0.018
Ancestry	English	312	565	0.552
	Chinese	20	565	0.035
	German	7	565	0.012
	Indian	10	565	0.018
	Australian	98	565	0.173
	Other	118	565	0.209
Occupational Status	Full-Time Employed	271	490	0.553
	Part-Time Employed (<34 Hours Per Week)	77	490	0.157
	Student	64	490	0.131
	Trainee	2	490	0.004
	Not Currently Working	32	490	0.065
	On Temporary Leave	5	490	0.010
	Other	39	490	0.080
Highest Level of Completed Education	Professional Degree (i.e. MD, JD etc completed)	10	489	0.020
	PhD Degree (Completed)	17	489	0.035
	Postgraduate Studies (MSc Completed)	67	489	0.137
	Undergraduate University Course (completed)	176	489	0.360
	Intermediate between secondary level and university (e.g. Technical training)	97	489	0.198
	Senior secondary school (Years 11 to 12)	101	489	0.207
	Secondary school (Years 7 to 10)	9	489	0.018
	Other	12	489	0.025
Living with:	Family of origin (two parents/partners, only child)	34	564	0.060
	Family of origin (two parents/partners and siblings)	108	564	0.191
	Mother (only child, parent divorced-separated-widowed)	19	564	0.034
	Mother and sibling(s) (parent divorced-separated-widowed)	17	564	0.030
	Father (only child, parent divorced-separated-widowed)	6	564	0.011
	Father and sibling(s) (parent divorced-separated-widowed)	5	564	0.009
	With Partner	149	564	0.264
	Alone	61	564	0.108
	With Friend(s)	28	564	0.050
	Temporary accommodation	4	564	0.007
	Other	18	564	0.032
	With Partner and Children	115	564	0.204
Relationship Status	Single	148	490	0.302
	In a Romantic Relationship	157	490	0.320
	Engaged	24	490	0.049
	Married	145	490	0.296
	De-facto	16	490	0.033
Social Media Usage	Yes	550	565	0.973
	No	15	565	0.027
Best Friend in Fav. Soc. Med	No	189	565	0.335
	Yes	376	565	0.665
Offline Friends in Fav. Game	No	312	565	0.552
	Yes	253	565	0.448
Offline Friends in Fav. Soc. Media	No	154	565	0.273
	Yes	411	565	0.727
Family Member in Fav. Game	No	406	565	0.719
	Yes	159	565	0.281
Family Member in Fav. Soc. Media	Yes	472	564	0.837
	No	92	564	0.163
Best Friend in Fav. Game	No	336	565	0.595
	Yes	229	565	0.405

*Note.* H_a_ is proportion ≠ 0.5.

**Table 12:** Parameterisation of Variance-Covariance Structures, From the Most to the Least Restrictive.

Model	Variances	Covariances	Parameterisation Type
1	Equal	Fixed to 0	Class-invariant diagonal parameterisation model (CIDP). This model presumes that relationships across model indicators should not be estimated (covariances fixed at zero) and that different profiles will exhibit qualitative similarities (equal variances).
2	Varying	Fixed to 0	Class-varying diagonal parameterisation model (CVDP). This model presumes that relationships between model indicators should not be estimated (covariances fixed at zero), and that different profiles will exhibit qualitative differences (varying variances).
3	Equal	Equal	Class-invariant unrestricted parametrisation model (CIUP). This model allows indicators to co-vary within profiles, and imposes restrictions that the variances and covariances must be equal across different profiles.
4	Varying	Varying	Class varying unrestricted parameterisation (CVUP). This model permits all indicators to co-vary within profiles, and it allows for different variances and covariances (i.e., residual correlations) across profiles. In essence, this model assumes that there are relationships between model indicators both within and between latent profiles that need to be estimated (i.e., varying covariances), and that different profiles will exhibit qualitative differences (varying variances).

*Note.* In the given context, “diagonal” implies that the sum of the elements in the variance-covariance matrix is zero, thereby preventing the model from estimating covariances between indicators

**Table 13:** Description of Flow Profiles, Including Participant Proportion.

Profile	*N*	*%*
Profile 1: High Flow with High Loss of Control (HF-HLOC)	79	14.0
Profile 2: Low Flow with Low Enjoyment (LF-LE)	67	11.9
Profile 3: Average Flow with Low Enjoyment (AF-LE)	99	17.5
Profile 4: Low Flow with High Enjoyment (LF-HE)	113	20.0
Profile 5: High Loss of Time with Low Loss of Control (HLOT-LLOC)	207	36.6

## Data Availability

The data has been made available

## Appendix B

**Data Collection Procedures**

***Step 1 (engagement):*** All potential participants and/or their parents/guardians and school principals, for those under age, will be sent an email with a link to the information to participants and consent forms. Those who wish to receive further information about the study, or would like to discuss the study with the researchers, will contact the researchers via email or phone. Information requested will be provided with the email, phone and/or over an arranged online video-call, as per participant's preference.

***Step 2 (consent):*** Those who wish to take part in the research will sign the consent form. For those <18 years, both parents/guardians and child will need to sign.

***Step 3 (anonymizing-pairing data):*** Each participant will receive an email including an anonymous research participation code and will be requested to indicate a date and time for a first face to face meeting with a member of the research team in an allocated space (i.e. consultation room) for the research at either the school premises or the Victoria University. One's research participation code will be used in all the data collection and the “key” for the code will be placed separated from the data (i.e., a record that pairs each participant's identity to their unique participation code, accessed via security codes possessed by the chief investigator). The participant's code will be used to match all data collection across the four time points (i.e., mobile data/Aware app, Fitbit data and questionnaires). A participant's code will be composed by the prefix “Fitbit”, followed by a number randomly attached to the participant in the study (e. g. fitbit1). Each participant's code will be requested to be provided at the beginning of their survey responses, during the installation of a mobile application called “Aware” on their phones, and before starting to wearing a physical activity bracelet tracker, called Fitbit (i.e. prior to be given to the participants, each Fitbit device gets coded with a unique number).

***Step 4 (data collection initiation-testing session):*** A face-to-face meeting between members of the research team and the participant will then occur at the predefined space and time (see step 3). In this meeting, a Fitbit watch will be fitted on the participant's wrist and the Aware mobile application will be installed on the participant's phones, while concurrently being paired with one's unique code. It is noted that the Aware app is a monitoring tool that records one's selected/approved aspects of mobile phone usage (i.e., one needs to approve during the installation of the app any specific form of monitoring enabled; e.g. screen on time). A Fitbit watch is a device that collects data related to the participants physical activity and sleep duration [21]. Participants will be encouraged/requested to keep these monitoring devices for a week's duration. During this time, their total steps, distance moved, calorie expenditure, sleep duration and active minutes (e.g., a measure of one's energy consumed while physically active [22]) will be monitored, alongside the number and duration/length of their calls/messages. The Aware app and the Fitbit monitoring can be interrupted or reviewed by the participant at any point they wish (e.g., one can delete the app, disable certain app monitoring features [e.g. frequency of texts received] and/or stop wearing the Fitbit). It is noted that this methodology has been approved for several research projects by the Institutional Review Boards (IRB) from other universities in Europe, the U.S, and the University of Melbourne in Australia [20]. During this first meeting, the online survey link will be also sent to the participant's email, to also address within seven days. At the end of this meeting, participants and research team members will arrange the time/date (as per meeting 1) that the Fitbit will be returned (after being carried for 7 days).

***Step 5 (data storage):*** The survey responses and the Aware data will be directly/automatically stored online in secured spaces on the VU server created by the VU IT. The Fitbit app sends each specific bracelet's records directly to an accountless email address created by the VU IT team (e.g. fitbit1@vu.edu.au) and hosted on the aforementioned server in a process advised by the VU IT team due to issues of compatibility. The research team can access the Fitbit data via this mailbox and then match it with the rest of one's data stored on the VU server. This will enable the de-identified matching of the participants' survey-responses, their Fitbit and their mobile “Aware” data collected every six months via the anonymous code supplied in step 3.

***Step 6 (data collection completion):*** Research members and participants will meet at the pre-arranged space and time to return the Fitbit device and de-activate the aware app after the seven days data collection period is due per time point (see steps 4 & 5). Steps 4, 5 and 6 will be repeated at the 6, 12- and 18-months data collection points.

Considering one's survey responses in particular, an online survey platform will be automatically set on the VU server, to send a link to the participants at the study start point and at 6-, 12-, and 18-months follow-up points (if one withdraws from the study their follow up links will be de-activated). The link will open a battery of sociodemographic questions and the questionnaire items. All questionnaires were adapted for use in Australia and two versions of the battery were made – one for adolescents (see attached adolescent form, Appendix B) and one for adults (see attached adult form, Appendix C) – as some of the questions were not applicable to both groups. Data will be securely (with the use of codes possessed by the chief investigator) accessed to proceed with the analyses (step 5).

See figures 2.1 and 2.2. for a graphical representation of this process.
